# Deciphering *Trifolium pratense* L. holobiont reveals a microbiome resilient to future climate changes

**DOI:** 10.1002/mbo3.1217

**Published:** 2021-07-23

**Authors:** Sara Fareed Mohamed Wahdan, Benjawan Tanunchai, Yu‐Ting Wu, Chakriya Sansupa, Martin Schädler, Turki M. Dawoud, François Buscot, Witoon Purahong

**Affiliations:** ^1^ Department of Soil Ecology UFZ‐Helmholtz Centre for Environmental Research Halle (Saale) Germany; ^2^ Department of Biology Leipzig University Leipzig Germany; ^3^ Botany Department Faculty of Science Suez Canal University Ismailia Egypt; ^4^ Department of Forestry National Pingtung University of Science and Technology Pingtung Taiwan; ^5^ Department of Community Ecology UFZ‐Helmholtz Centre for Environmental Research Halle (Saale) Germany; ^6^ German Centre for Integrative Biodiversity Research (iDiv) Halle‐Jena‐Leipzig Leipzig Germany; ^7^ Botany and Microbiology Department College of Science King Saud University Riyadh Saudi Arabia

**Keywords:** climate change, plant endosphere, Illumina Miseq, microbiome, rhizosphere, *Trifolium pratense*

## Abstract

The plant microbiome supports plant growth, fitness, and resistance against climate change. *Trifolium pratense* (red clover), an important forage legume crop, positively contributes to ecosystem sustainability. However, *T*. *pratense* is known to have limited adaptive ability toward climate change. Here, the *T*. *pratense* microbiomes (including both bacteria and fungi) of the rhizosphere and the root, shoot, and flower endospheres were comparatively examined using metabarcoding in a field located in Central Germany that mimics the climate conditions projected for the next 50–70 years in comparison with the current climate conditions. Additionally, the ecological functions and metabolic genes of the microbial communities colonizing each plant compartment were predicted using FUNGuild, FAPROTAX, and Tax4Fun annotation tools. Our results showed that the individual plant compartments were colonized by specific microbes. The bacterial and fungal community compositions of the belowground plant compartments did not vary under future climate conditions. However, future climate conditions slightly altered the relative abundances of specific fungal classes of the aboveground compartments. We predicted several microbial functional genes of the *T*. *pratense* microbiome involved in plant growth processes, such as biofertilization (nitrogen fixation, phosphorus solubilization, and siderophore biosynthesis) and biostimulation (phytohormone and auxin production). Our findings indicated that *T*. *pratense* microbiomes show a degree of resilience to future climate changes. Additionally, microbes inhabiting *T*. *pratense* may not only contribute to plant growth promotion but also to ecosystem sustainability.

## Graphical abstract

The *Trifolium pratense* microbiomes (including both bacteria and fungi) of the rhizosphere and the root, shoot, and flower endospheres were comparatively examined using metabarcoding in a field located in Central Germany that mimics the climate conditions projected for the next 50–70 years in comparison with the current climate conditions. Our findings indicated that *T. pratense* microbiomes show a degree of resilience to future climate changes. Additionally, microorganisms inhabiting *T. pratense* may not only contribute to plant growth promotion but also to ecosystem sustainability.

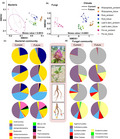

## 1 INTRODUCTION

Forage legume crops with high protein and fiber contents are a major livestock feed source. The integration of forage legumes into the cropping systems can have beneficial effects on soil health and fertility, as well as on controlling weeds, insect pests, and pathogens (Sheaffer & Seguin, 2008). *Trifolium pratense* L. (red clover), a forage legume crop in the temperate regions, is a key component of sustainable livestock farming systems (De Vega et al., 2015). In the 16^th^ century, *T*. *pratense* was used as a protein‐rich fodder in livestock agriculture. *T*. *pratense* was further used as a “nitrogen‐assimilating crop” in the 19^th^ century when the soil nitrogen content depleted in Europe (Kjærgaard, 2003; McKenna et al., 2018). Red clover efficiently fixes atmospheric nitrogen (N) due to its symbiotic association with N‐fixing bacteria (Fustec et al., 2010). Additionally, the use of red clover increases soil fertility through the rhizodeposition of plant exudates containing soluble N compounds (Paynel et al., 2008). The decomposition of red clover residues results in the release of 40–70% of the total plant N into the soil within 5–10 weeks of decomposition (Lupwayi et al., 2006). Therefore, red clover is considered a “fertility‐building crop” (McKenna et al., 2018). The incorporation of red clover in agricultural crop rotations is an alternative and sustainable method of introducing N into low‐input agricultural practices. In addition to its application in agriculture, red clover has pharmacological applications as it exhibits oestrogenic, antispasmodic, and expectorant properties (Coon et al., 2007; Leung & Foster, 1996; Lin et al., 2000).

The events associated with climate change, including increased global temperatures and altered precipitation patterns, adversely affect plant health and productivity across different agroecosystems (Franklin et al., 2016; Schmidhuber & Tubiello, 2007). Recent studies have suggested that climate change has led to shifts in plant phenology, species distribution, and population dynamics and has contributed to the emergence of new potential fungal plant pathogens (Delgado‐Baquerizo et al., 2020; Franklin et al., 2016; Wahdan et al., 2020). *T*. *pratense* is adapted to a wide range of soil types and pH levels in temperate regions. However, it has a limited capacity to adapt to increased temperatures and extreme drought events (Hanna et al., 2018). Previous studies have reported that red clover is resistant to a maximum temperature of 25°C but that prolonged exposure to 28°C decreases the crop yield (Hanna et al., 2018). Additionally, red clover exhibited some resistance to moderate drought, however, extreme drought highly impaired the yield that did not recover after a post‐drought period (Hofer et al., 2016). Various studies have examined the cascading effects of climate change on *T*. *pratense* performance. However, the response of the *T*. *pratense* microbiome to climate changes has not been examined.

The plant holobiont, which comprises the host plant and its endocellular and extracellular microbiome (Rosenberg & Zilber‐Rosenberg, 2018), is considered a biological entity associated with stability, adaptation, and evolution, and not as individual biotic components (Vandenkoornhuyse et al., 2015). The host plant traits, such as resistance against pathogens, immune system priming, and growth, are dependent on the host's microbiome composition (Hartmann et al., 2007; Mendes et al., 2011; Ritpitakphong et al., 2016; Trivedi et al., 2020). In contrast to the highly conserved plant genome, the microbiome genome is prone to rapid genetic changes (Rosenberg & Zilber‐Rosenberg, 2018). Therefore, the plasticity of the microbiome to adapt to environmental changes enables rapid host adaptation (Voolstra & Ziegler, 2020). Microbiome plasticity is a broad phenomenon that includes a dynamic reconstruction of microbiome composition by increasing and/or decreasing the abundance of specific microbes and/or by the colonization of novel microbes that facilitate the host adaptation to external stress (Bulgarelli et al., 2013; Haney et al., 2015). However, enhanced microbiome plasticity increases the risk of pathogen invasion and undesirable microbes enrichment with a concomitant loss of beneficial ones (Voolstra & Ziegler, 2020). Beneficial microbiome plasticity depends on the dynamics within useful microbes that maintain high levels of functional redundancy in the original microbial communities. In another scenario, the microbiome may respond to environmental changes by exhibiting resistance or by maintaining a constant community structure with a high potential to adapt to external stress (Allison & Martiny, 2008). The plasticity or resistance of host‐associated microbiomes may contribute to host adaptation. Nevertheless, the adaptive strategies employed by the *T*. *pratense* microbiome in response to future climate conditions are so far unclear.

Within the host plant, microbial communities vary between the belowground and aboveground plant compartments, which are distinct ecological niches with variations in nutrients and oxygen availability in different tissue types (Beckers et al., 2017; Cregger et al., 2018; Pangesti et al., 2020; Zarraonaindia et al., 2015). Microorganisms reach their host to form the indigenous microbiome through the following two pathways: vertical transmission via seeds and horizontal transmission from the surrounding atmosphere, rhizosphere, and bulk soils (Trivedi et al., 2020). The rhizosphere is the soil zone around the roots in which microbes are impacted by the presence of plant roots (Vandenkoornhuyse et al., 2015). The density of microbial populations in the rhizosphere is higher than that in the bulk soils; therefore, it is considered a hot spot for plant‐microbiome interaction (Berendsen et al., 2012). The microbiome composition of the root endosphere depends on the ability of microbes to invade root tissues from the surrounding rhizosphere and rhizoplane (Pangesti et al., 2020; Vandenkoornhuyse et al., 2015). Soil is also a microbial reservoir for the aboveground plant compartments as some endophytic microbes (microbes that colonize the internal plant tissue showing no infection or negative effect on their host; Schulz and Boyle (2006)) of the aboveground plant compartments/niches are recruited from soil (Zarraonaindia et al., 2015). Additionally, the aboveground endophytic microbiomes are derived from microbes that first colonize the leaf and flower surfaces as epiphytes (Vandenkoornhuyse et al., 2015) and can passively or actively invade the plant tissues (De Vrieze et al., 2018).

The ability of the host plants to utilize beneficial microbes, such as plant growth‐promoting bacteria (PGPB) determines their response to the environmental and climate changes through direct and indirect mechanisms. These mechanisms include nutrient solubilization, biological nitrogen fixation, and the production of plant growth regulators, organic acids, and volatile organic compounds (Ahkami et al., 2017). Therefore, the identification of the *T*. *pratense* microbiome functional profile is critical for developing new strategies to enhance plant health, growth, and resistance against future climate changes.

This study aimed to investigate the responses of the bacteriome and mycobiome (i.e., bacterial and fungal microbiomes) associated with four ecological niches/compartments of *T*. *pratense* and to evaluate their potential ecological and metabolic functions in responding to future climate conditions. The rhizosphere and the endospheres of the root, shoot system (leaves and stems), and flower were examined. The study was performed at grassland plots of the Global Change Experimental Facility (GCEF) established in central Germany (Schädler et al., 2019). GCEF is one of the largest experimental platforms designed to investigate the effect of a future climate scenario mimicking the prediction for the next 50–70 years on ecosystem processes in plots under different land‐uses (Schädler et al., 2019). The sampling was performed 4 years after starting the climate manipulation in summer as it represents the critical season in which the future climate scenario is expected to have the highest impacts on soil functions (Yin et al., 2019). The period of 4 years after the onset of the experiment was sufficient for *T*. *pratense* generation and their microbiome to be affected by climate manipulation and adapt through the vertical and horizontal transmission of new microorganisms. MiSeq Illumina sequencing of the 16S rRNA gene (V5–V7 region) and the nuclear ribosomal internal transcribed spacer region 2 (ITS2) was performed to characterize the bacterial and fungal microbiomes, respectively. We hypothesized that *T*. *pratense*‐associated microbiomes would be shaped by the influence of both biotic (plant compartments/ecological niches) and abiotic (climate change) factors that varied in their relative importance.

## 2 MATERIALS AND METHODS

### 2.1 Study site and experimental design

**FIGURE A1:**
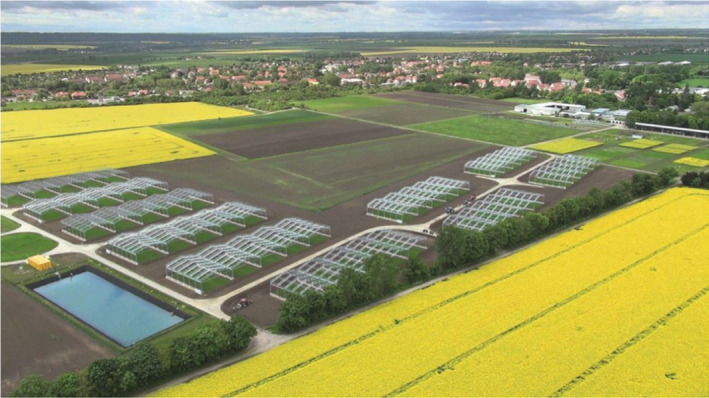
Aerial view for the Global Change Experimental Facility (GCEF) field research station of the Helmholtz Centre for Environmental Research in Bad Lauchstädt, Saxony‐Anhalt, Germany, photograph taken by Tricklabor Berlin/Service Drone

**FIGURE A2:**
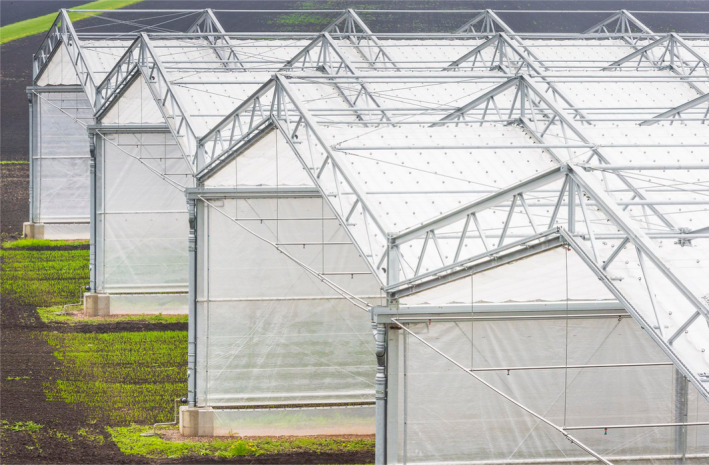
Closed shelters and panels of the future climate plots of the GCEF, photograph taken by UFZ/ André Künzelmann

**FIGURE A3:**
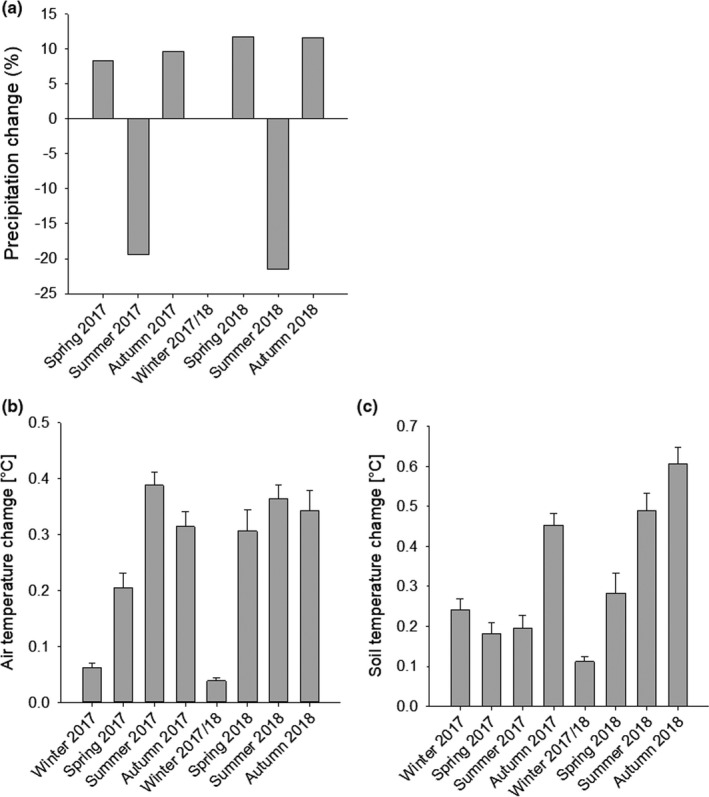
Effects of climate manipulation on (a) total precipitation (sum of seasons), (b) air temperature in a height of 10 cm (daily mean temperature + standard error), and (c) soil temperature (daily mean temperature + standard error) in a depth of 1 cm in experimental plots managed as extensively used grassland in the GCEF. Precipitation is not manipulated during the winter months. Note that the effects of soil temperature are strongly modulated by indirect effects via the change of vegetation cover (see also Schädler et al., 2019). Thus, the values presented here, are the net result of the direct increase of temperature manipulation and the indirect modulation by the vegetation cover.

The study was conducted in GCEF at the field research station of the Helmholtz Centre for Environmental Research in Bad Lauchstädt, Saxony‐Anhalt, Germany (51_22060 N, 11_50060 E, 118 m a.s.l.). The area is characterized by a subcontinental climate (mean temperature, 8.9°C and mean annual rainfall, 498 mm for the period 1896–2013; mean temperature, 9.8°C and mean annual rainfall, 516 mm for the period 1995–2014). During the study period (2018), the mean temperature was 10.8°C with an annual rainfall of 254 mm. The study field comprised the Haplic Chernozem soil, which was characterized by a high content of organic carbon till a depth of more than 40 cm and a high water‐holding capacity (Altermann et al., 2005). The GCEF field infrastructure (Figure A1) was designed to comparatively investigate the consequences of future climate and current climate conditions on ecosystem processes in different land types (Schädler et al., 2019). Furthermore, the GCEF comprises 50 field plots (400 m^2^ each), which were equally divided and subjected to the current and future climate conditions. Future climate condition is a consensus scenario across three models (COSMO‐CLM (Rockel et al., 2008), REMO (Jacob & Podzun, 1997), and RCAO (Döscher et al., 2002)) of climate change in Central Germany for the years 2070–2100. Hence, future climate plots (Figure A2) are equipped with mobile shelters and side panels, as well as an irrigation system. The roofs are controlled by a rain sensor. The continuous adjustment of irrigation or roof closing has decreased the precipitation by approximately 20% in the summer months and increased the precipitation by approximately 10% in spring and autumn. To simulate the increase in temperature, the standard method “passive night‐time warming” was used. The shelters and panels were automatically closed from sundown to sunrise to increase the mean daily temperature by approximately 0.55°C accompanied by a stronger increase in minimum temperatures (up to 1.14°C on average). Current climate plots are equipped with the same steel constructions (but without shelters, panels, and irrigation systems) to mimic the possible microclimatic effects of the experimental setup. The resulting changes in climate conditions, due to climate manipulation, before and during the study period are shown in Figure A3. For more details on the field station design, see Schädler et al. (2019). The experiment was performed in the extensively used meadow plots subjected to future climate conditions (5 plots) in comparison with the plots of current climate conditions (5 plots). The vegetation comprises 56 plant species that were chosen from multiple regional natural source populations located in Central Germany. Each source population is genetically different. *T*. *pratense* species is represented by 2 gene pools (Madaj et al., 2020). The vegetation was mowed twice a year without the application of herbicides or fertilizers. The experiment was conducted in mid‐July 2018 (summer), which corresponded with the highest effect of future climate conditions on soil ecosystem function (plant residue decomposition) at the GCEF in other years (Yin et al., 2019).

### 2.2 Sample collection and compartmentalization of the belowground and aboveground plant compartments

Each climate scenario was represented by five plots. At each plot, three healthy *T*. *pratense* L. (red clover) plants were randomly selected and their two belowground compartments (rhizosphere soil and root) and two aboveground compartments (leaf/stem and flower) were examined. In total, 30 plants (3 plants × 10 plots) were sampled, the two halves of which are representing current and future climate scenarios. For each plant, the bulk soil was removed by vigorous shaking for 10 min. The adhering rhizosphere soil was collected by vortexing the roots for 10 min in a sterile polymerase chain reaction (PCR) water (Barillot et al., 2012). The root was separated from the aboveground compartments and surface‐sterilized to collect the endophytes. Briefly, the root was washed under running distilled water, followed by three washes with 0.1% Tween 20, a 3 min wash with 70% ethanol, and five washes with sterilized distilled water. Similarly, the endophytes were obtained from the aboveground compartments after surface sterilization. The leaves and stems were considered as one compartment, while the flowers were considered a separate compartment. The two compartments were washed twice with 0.1% Tween 20, followed by five washes with sterilized distilled water. The samples from the three plants of each plot were pooled into a single composite sample. The entire sterilized compartments (root, leaves/stems, and flowers) were crushed using liquid nitrogen and the resulting powder was used for DNA extraction.

### 2.3 DNA extraction, amplicon library preparation, and Illumina MiSeq sequencing

The DNA extraction was carried out using 250 mg of each plant compartment and rhizosphere sample using the DNeasy PowerSoil kit™ (Qiagen Inc.), following the manufacturer's instructions and subjected to PCR. The V5–V7 region of the bacterial 16S rRNA was amplified using the following primers: BAC799F forward (5′‐AACMGGATTAGATACCCKG‐3′) (Chelius & Triplett, 2001) and BAC1193R reverse (5′‐ACGTCATCCCCACCTTCC‐3′) (Bodenhausen et al., 2013). These bacterial primer pairs were chosen because they do not amplify the chloroplast DNA (Beckers et al., 2016). The ITS2 region of fungi was amplified using the following primers: fITS7F forward (5′‐GTGARTCATCGAATCTTTG‐3′) (White et al., 1990) and ITS4 reverse (5′‐TCCTC CGCTTATTGATATGC‐3′) (White et al., 1990). The amplification was performed in a two‐step process. The forward primer of the first PCR was constructed using the Illumina i5 sequencing primer (5′‐TCGTCGGCAGCGTCAGATGTG TATAAGA GACAG‐3′) and a specific forward primer. The reverse primer was constructed using the Illumina i7 sequencing primer (5′‐GTCTCGTGGG CTCGGAGATGTGTATAAGAGACAG‐3′) and the specific reverse primer. The amplification was performed in a 25 μl reaction volume comprising 1 μl (5 μM) of each primer and 1 μl of the template using the Qiagen HotStar hi‐fidelity polymerase kit (Qiagen Inc.). PCR was performed using an ABI Veriti thermocycler (Applied Biosystems). The PCR conditions were as follows: 95°C for 5 min, followed by 35 cycles of 94°C for 15 s, 54°C for 60 s, and 72°C for 1 min and one step of 72°C for 10 min and 4°C hold. The amplicons from the first PCR, whose concentrations were quantitatively determined, were used for the second PCR. In the second PCR, dual indices were attached using the Nextera XT index kit. The conditions for the second PCR were the same as those used for the first PCR, except for the amplification cycles (10 amplification cycles used in the second PCR). The amplicons were visualized using eGels (Life Technologies), following the manufacturer's instructions. Equimolar concentrations of the products were pooled, and the size of each pool was selected in two rounds using Agencourt AMPure XP (BeckmanCoulter) in a 0.75 ratio for both rounds. The size‐selected pools were then quantified using a Quibit 2.0 fluorometer (Life Technologies). Sequencing was performed using MiSeq (Illumina, Inc) with a 2 × 300 bp paired‐end strategy, following the manufacturer's instructions.

### 2.4 Processing of amplicon data

The primer sequences were trimmed from the demultiplexed raw reads using cutadapt (Martin, 2011). The pair‐end raw reads of bacterial and fungal datasets were merged using the simple Bayesian algorithm with a threshold of 0.6 and a minimum overlap of 20 nucleotides as implemented in PANDAseq (Masella et al., 2012). All the assembled reads were filtered for high‐quality sequence reads (minimum sequence length, 350 and 120 nucleotides for bacteria and fungi, respectively; maximum sequence length, 500 and 580 nucleotides for bacteria and fungi, respectively; minimum average Phred score of 25; maximum length of 20 homopolymers in the sequence and without ambiguous nucleotides). Potential chimeras were removed using UCHIME (Edgar et al., 2011) as implemented in MOTHUR (Schloss et al., 2009). The high‐quality reads were clustered into operational taxonomic units (OTUs) using cd‐hit‐est 4.6.2 (Fu et al., 2012) at a threshold of 97% pairwise similarity. The bacterial 16S rRNA OTU representative sequences were assigned against the SILVA v132 reference sequence database (Quast et al., 2013) to obtain the respective OTU tables. Fungal ITS representative sequences were assigned against the UNITE v7 sequence database (Kõljalg et al., 2013) using the Bayesian classifier as implemented in MOTHUR (Schloss et al., 2009). Singleton and doubleton OTUs originating from sequencing errors were removed from the datasets. The sequences that were classified as “Cyanobacteria,” “Chloroplast,” or “Mitochondria” and those that were not classified at the kingdom level were removed from the bacterial dataset. The ecological and metabolic functions of bacterial OTUs were predicted using FAPROTAX (Louca et al., 2016) and the functional annotation tool of prokaryotic taxa v.1.1, whereas those of fungal OTUs were predicted using FUNGuild (Nguyen et al., 2016). Additionally, the Tax4Fun (Aßhauer et al., 2015) R package, which employs 16S rRNA gene‐based taxonomic information, and the Kyoto Encyclopaedia of Genes and Genomes (KEGG) database were used to predict the metabolic functional attributes of bacterial communities in the rhizosphere and endosphere of *T*. *pratense*. Tax4Fun converted the SILVA‐labeled OTUs into prokaryotic KEGG organisms and normalized these predictions using the 16S rRNA copy number (obtained from the National Center for Biotechnology Information genome annotations).

### 2.5 Physicochemical analyses of the rhizosphere soil

The rhizosphere soil samples (100–200 g wet weight) from each plot were dried and sieved. The pH of the rhizosphere soil was measured using WTW Multi 3510 IDS. The rhizosphere soil was subjected to dry combustion at 1000°C to determine the total carbon (TC) and total nitrogen (TN) concentrations using a CHNS‐Elemental Analyzer (Elementar Analysensysteme GmbH), following the manufacturer's instructions. Soil carbon/nitrogen (C/N) stoichiometry was calculated based on TC and TN. Available soil phosphorus was extracted and measured according to the Bray 1 method (Gutiérrez Boem et al., 2011). Cations (K^+^, Mg^2+^, Ca^2+^, and Na^+^) in the rhizosphere soil were determined using an atomic absorption spectrophotometer (Hitachi Z 5300, Hitachi‐Science & Technology), following the manufacturer's instructions. Physicochemical properties of soil did not differ significantly between current climate and future climate plots (Table A1).

### 2.6 Statistical analysis

All statistical analyses were performed using the PAST program version 2.17c (Hammer et al., 2001) and R environment version 3.6.1 (R‐Development‐Core‐Team, 2019). All the analyses were conducted based on five independent replicate plots of the field experiment (*n* = 5) for each treatment. The datasets were normalized to the minimum number of sequence reads per sample (5360 and 10,338 sequence reads for bacterial and fungal OTUs, respectively) using the function “rrarefy” from the vegan (Oksanen et al., 2019) package in the R environment version 3.6.1 (R‐Development‐Core‐Team, 2019). To provide an overview of the bacterial and fungal operational taxonomic units (OTUs) distribution among different plant compartments, the shared and unique OTUs were represented using a Venn diagram with the software available at http://bioinformatics.psb.ugent.be. The microbial diversity indices (Simpson's diversity, observed OTU richness, and estimated richness (Chao‐1)) were calculated for both bacteria and fungi. Variance homogeneity was examined using Levene's test. The normal distribution of data was examined using the Shapiro–Wilk test. Since some samples’ diversity was skewed, we used log_10_‐transformed diversity indices data for further statistical analysis while the original values were used only for data visualization (Figure 2). To test the influence of climate, plant compartment, and their interaction on microbial diversity, a split‐plot analysis of variance (ANOVA) was performed using the function “sp.plot” from the agricolae R package (de Mendiburu, 2016). In detail, the impact of climate (two levels) was analyzed at the main‐plot level, while that of the plant compartment (four levels) and both plant compartment and climate were analyzed at the sub‐plot level. Based on split‐plot ANOVA results, the least significant difference (LSD) test was applied, using the function ‘LSD.test’, to show differences between treatments.

Microbial (bacteria and fungi) community composition was assessed by computing Jaccard and Bray–Curtis dissimilarity matrices and then visualized using non‐metric dimensional scaling (NMDS) ordinations using the function “metaMDS” in the vegan R package (Oksanen et al., 2019) to visualize compositional differences. To test whether ecological niche (plant compartment), climate, or their interaction had a significant effect on community composition, permutational multivariate analysis of variance (NPMANOVA) (Anderson, 2001), and analysis of similarities (ANOSIM) based on Bray–Curtis and Jaccard dissimilarities between microbial communities (OTU level) were performed for 999 permutations. Additionally, NPMANOVA pairwise post hoc comparisons were performed to evaluate the effect of the tested factors on bacterial and fungal communities separately using the function “pairwise.adonis” in the vegan R package (Oksanen et al., 2019). Similarly, NPMANOVA, NMDS, and heat map were performed to test the impact of plant compartment, climate, or their interaction on the functional composition of microbes colonizing *T*. *pratense*. To assess the significant effect of plant tissue differentiation on the distribution of the most abundant microbial classes among the four plant compartments, the Kruskal–Wallis test was performed. Similarly, the Mann–Whitney U test was used to assess the significant influence of climate change on the relative abundance of each microbial class colonizing the same plant compartment. The hierarchical cluster analysis (HCA) was applied based on the Bray–Curtis dissimilarity matrix to test the plant niche‐specific and climate effect on the most abundant bacterial and fungal genera. Similarity percentages (SIMPER) analysis was performed with PAST software to examine the dissimilarities between the plant compartments. To determine which OTUs occurred more frequently between compartments (rhizosphere, root, leaf/stem, and flower) and climate (current *vs*. future), the indicator species analysis was performed using the function “multipatt” of the indicspecies R package (De Cáceres & Legendre, 2009). Before calculation of indicator species, component A (specificity; the probability that the sample belonged to the group after the species has been identified) and component B (sensitivity; probability of finding the species in samples belonging to the group), and to avoid the bias of low abundance OTUs, only OTUs that appeared with ≥0.001% relative abundance across all samples were chosen to perform the test. Only microbial endophytes were included, and no indicator species analysis was performed for the rhizosphere microbiome.

## 3 RESULTS

### 3.1 Richness and diversity of *T*. *pratense* microbiome under current and future climate conditions

**FIGURE 1:**
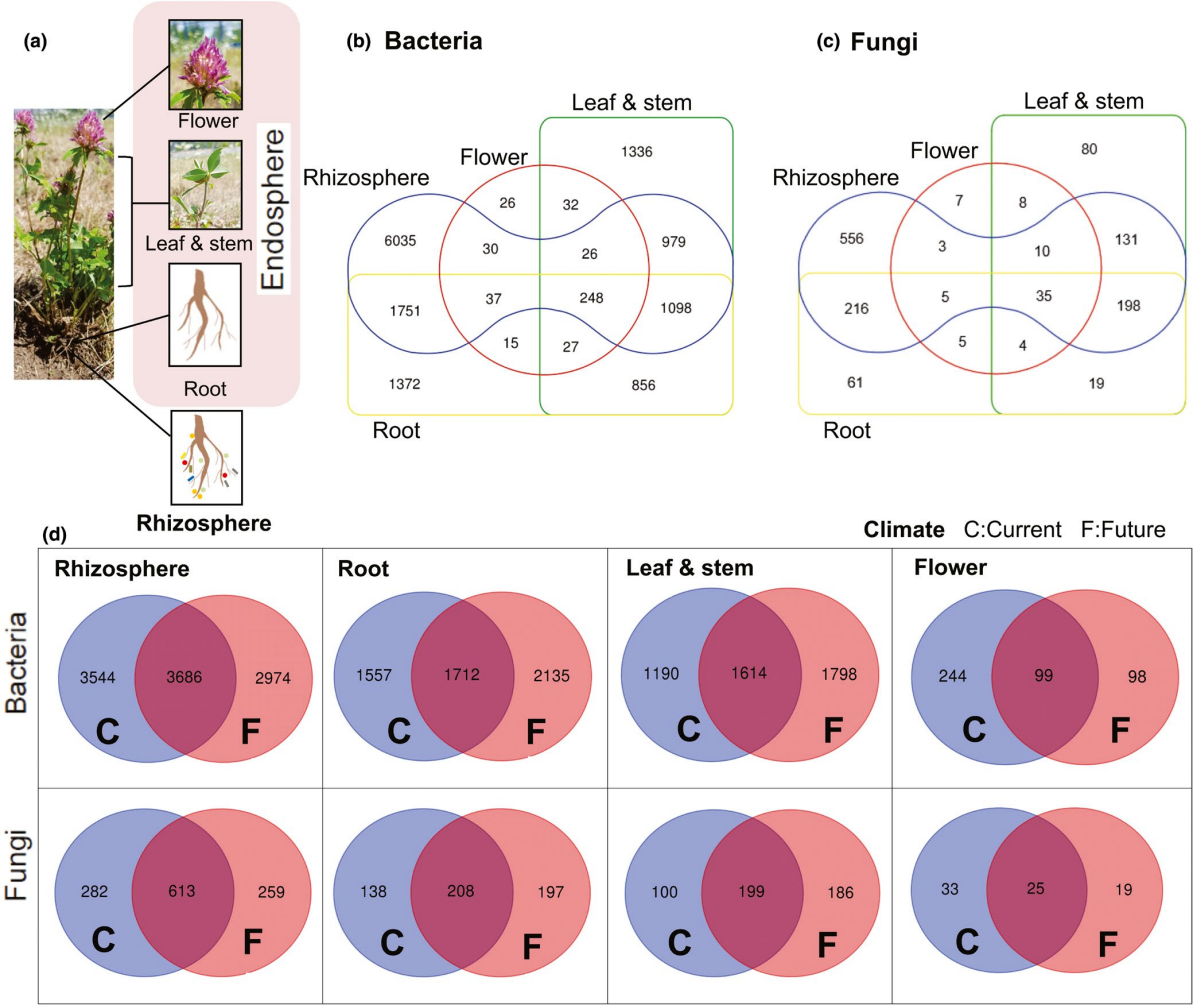
(a) Compartmentalization of *Trifolium pratense*. Venn diagrams showing the distribution of (b) bacterial and (c) fungal operational taxonomic units in each plant compartment and (d) climate conditions for each compartment

The distribution of bacterial and fungal OTUs in the plant compartments (rhizosphere and the root, leaf/stem, and flower endospheres) under both current and future climate conditions was analyzed (Figure 1). The rhizosphere soil harbored the highest number of unique OTUs (43.5% and 41.5% for bacteria and fungi, respectively). A large proportion of the OTUs in the rhizosphere was shared with the root (30.6% and 39% for bacteria and fungi, respectively), followed by leaf/stem (23% and 32.4% for bacteria and fungi, respectively), and flower (3.3% and 4.6% for bacteria and fungi, respectively) endospheres (Figure 1b,c). Only 1.7% of bacterial and 2.6% of fungal OTUs were shared among all compartments. The future climate condition‐specific OTUs were the highest for the root (40% and 36% of all bacterial and fungal OTUs, respectively) endosphere, followed by leaf/stem (39% and 38% of all bacterial and fungal OTUs, respectively) endosphere, rhizosphere (29% and 22% of all bacterial and fungal OTUs, respectively), and flower (22% and 25% of all bacterial and fungal OTUs, respectively) endosphere (Figure 1d).

**FIGURE 2:**
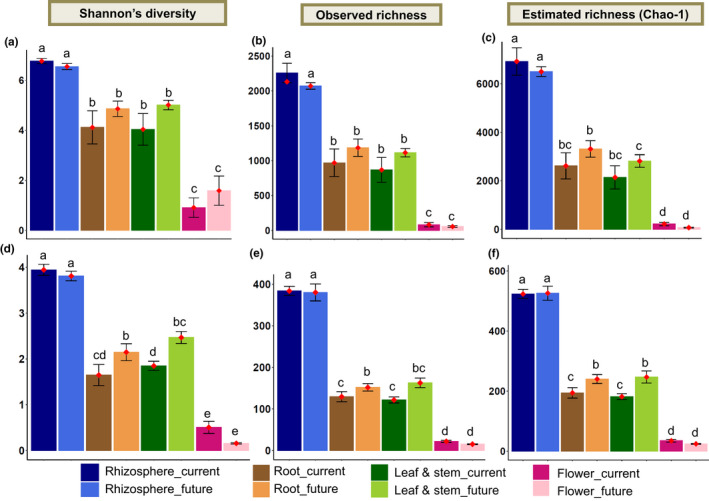
Alpha diversity indices of (a–c) bacterial and (d–f) fungal microbiomes in each compartment of *Trifolium pratense* under both current and future climate conditions. Error bars indicate the standard error; ♦ represent mean values. Different lower‐case letters indicate significant differences (*p* < 0.05) according to Fisher's Least Significant Difference

The effects of plant compartments, climate, or both on alpha diversity indices (Shannon's diversity, observed richness, and estimated richness) of *T*. *pratense* microbiomes were examined. Plant compartments had the highest influence on shaping microbial diversity and richness (Table 1). Climate change influenced the diversity and estimated richness of fungi (Table 1). Fungal diversity and richness were significantly higher in the leaf/stem and root endospheres under the future climate conditions compared to current climate conditions (Figure 2).

**TABLE 1:** Results of split‐plot analysis of variance of the effects of climate, plant compartment, or their interactions on bacterial and fungal diversity indices

Source of variation	Shannon's diversity			Observed richness			Estimated richness (Chao‐1)		
	df	*F* value	Pr (>F)	df	*F* value	Pr (>F)	df	*F* value	Pr (>F)
Bacteria									
Climate	1	1.49	0.289	1	0.532	0.506	1	0.978	0.378
Plant compartment	3	67.70	**<0.001**	3	115.39	**<0.001**	3	108.61	**<0.001**
Climate × Plant compartment	3	0.93	0.438	3	1.66	0.200	3	1.19	0.334
Fungi									
Climate	1	19.58	**0.011**	1	3.91	0.118	1	9.22	**0.038**
Plant compartment	3	194.18	**<0.001**	3	428.83	**<0.001**	3	397.76	**<0.001**
Climate × Plant compartment	3	5.15	**0.006**	3	2.346	*0.098*	3	2.98	*0.051*

Significant values (*p* < 0.05) are indicated in bold, marginal significant (*p* < 0.1) values are indicated in italic.

### 3.2 Community composition and taxonomic structure of *T*. *pratense* microbiome

**FIGURE 3:**
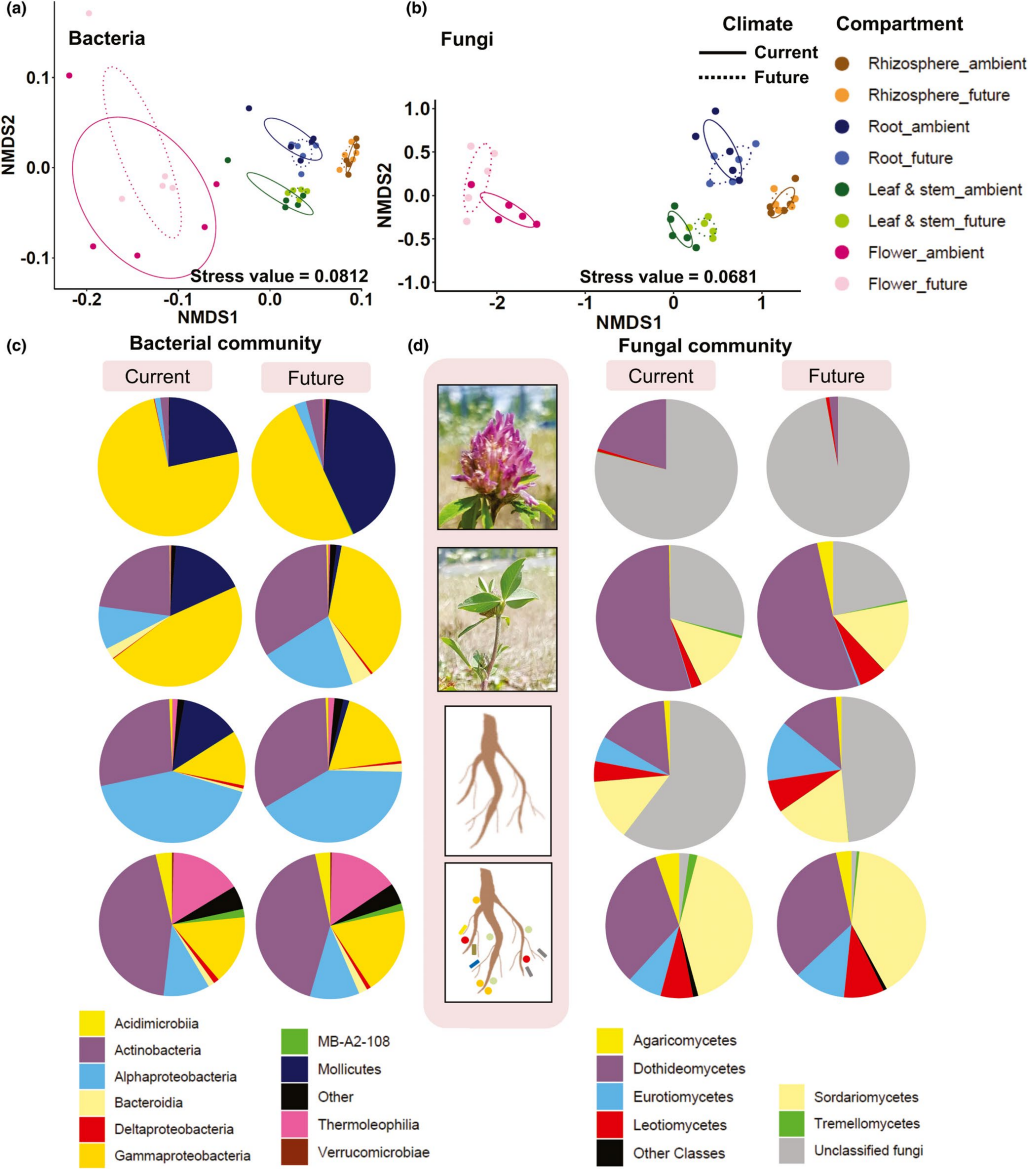
Community composition of *Trifolium pratense*. Nonmetric multidimensional scaling (NMDS) ordination of variation in the (a) bacterial and (b) fungal community structures of *T*. *pratense* in the individual plant compartments under current and future climate conditions. *T*. *pratense* was cultivated in the grassland ecosystem. The plot is based on Jaccard dissimilarities between microbial communities at the operational taxonomic unit level across 40 samples (permutations = 999). The samples (points) are shaded based on the plant compartment and climate conditions. Ellipses indicate a 95% confidence interval surrounding each group. Taxonomic composition (class level) of *T*. *pratense* (c) bacteriome and (d) mycobiome across individual plant compartments under current and future climate conditions. Illustrated classes are the most abundant (>1% relative abundance in each group) taxa

The composition of the bacterial and fungal microbiomes of *T*. *pratense* at the OTU level (97% identity) was examined. NPMANOVA corroborated by NMDS plots based on unweighted UniFrac distances (Figure 3a,b; Table A2) revealed that the microbial (both bacteria and fungi) communities distinctively clustered based on the plant compartments (bacteria, *F* = 8.68 and *p* = 0.001; fungi, *F* = 7.12 and *p* = 0.001) but not based on the climate conditions. Meanwhile, post hoc pairwise NPMANOVA revealed unique bacterial and fungal communities for each plant compartment (Tables A3 and A4). The analysis of Bray–Curtis distance revealed similar findings (Table A2).

Among the samples, 52 bacterial classes were detected. Of these, the abundance of 10 bacterial classes (>97% of total sequences relative abundance) significantly differed (except for Mollicutes) between the compartments (Figure 3c). The abundances of Actinobacteria (Kruskal–Wallis: *ꭓ* ^2^ = 25.95, *p* = 9.90 × 10^−6^) and Thermoleophilia (Kruskal–Wallis: *ꭓ* ^2^ = 26.49, *p* = 7.43 × 10^−6^) were significantly high in the rhizosphere as compared to other compartments, while those of Alphaproteobacteria (Kruskal–Wallis: *ꭓ* ^2^ = 27.14, *p* = 5.50 × 10^−7^) and Gammaproteobacteria (Kruskal–Wallis: *ꭓ* ^2^ = 13.17, *p* = 0.044) were significantly high in the root and leaf/stem endospheres as compared to other compartments. In total, 21 fungal classes were detected. Of these, the abundance of six classes, which accounted for more than 57% of the sequence relative abundance (Figure 3d), significantly differed among the plant compartments. The leaf/stem endosphere was significantly enriched in Dothideomycetes (Kruskal–Wallis: *ꭓ* ^2^ = 27.57, *p* = 4.47 × 10^−6^) and depleted in Eurotiomycetes (Kruskal–Wallis: *ꭓ* ^2^ = 33.07, *p* = 2.58 × 10^−7^). Meanwhile, Sordariomycetes (Kruskal–Wallis: *ꭓ* ^2^ = 32.71, *p* = 3.67 × 10^−7^) and Agaricomycetes (Kruskal–Wallis: *ꭓ* ^2^ = 27.28, *p* = 4.62 × 10^−6^) were significantly enriched in the rhizosphere compared to other compartments. Future climate conditions did not significantly affect the relative abundances of the dominant bacterial classes in the plant compartments. For fungi, the future climate conditions increased the relative abundance of Eurotiomycetes (Mann–Whitney: *p* = 0.015) and Agaricomycetes (Mann–Whitney *U* test: *p* = 0.031) in the leaf/stem endosphere and decreased the relative abundance of Tremellomycetes in the leaf/stem (Mann–Whitney *U* test; *p* = 0.023) and flower (Mann–Whitney *U* test; *p* = 0.039) endospheres.

**FIGURE A4:**
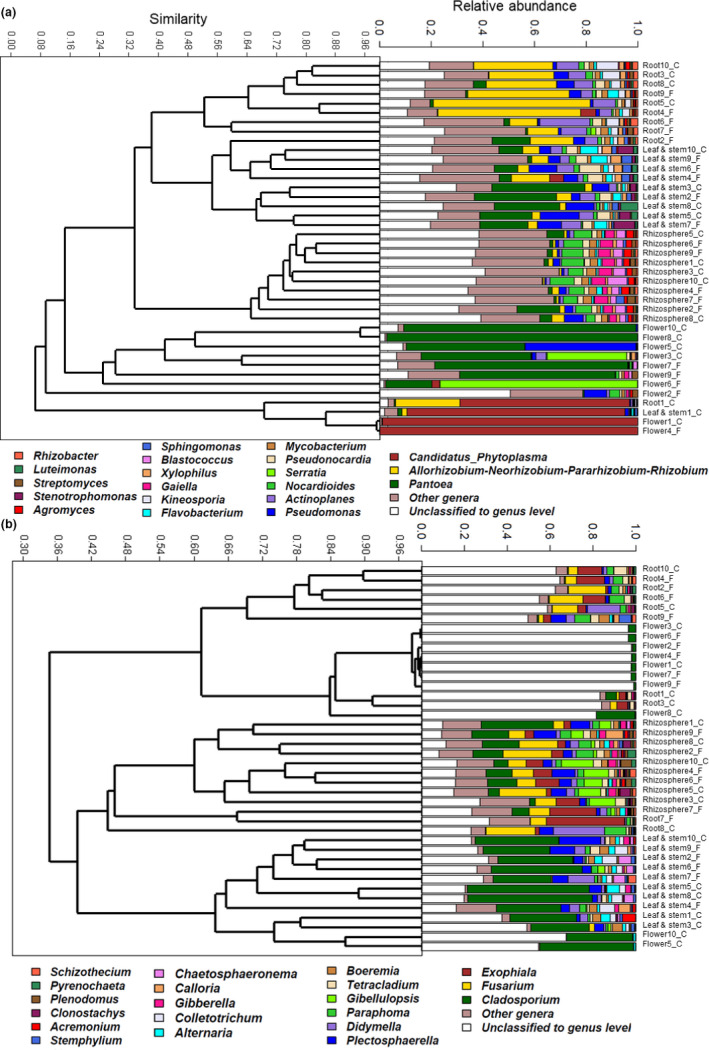
Hierarchical clustering and a bar plot of relative abundances of the most abundant (a) bacterial and (b) fungal genera among *Trifolium pratense* microbiome

Hierarchical clustering of the microbial community composition at the genus level revealed that the plant compartments were the major determinant of genera composition (Figure A4). The most abundant bacterial genera were *Pantoea* and *Rhizobia* (relative abundance of 16% and 9% among all bacterial sequences, respectively), while the most abundant fungal genera were *Cladosporium* and *Fusarium* (relative abundances of 15.6% and 5% among all fungal sequences, respectively). The compartment dissimilarity based on genera was calculated using SIMPER analysis (Table A5). *Allorhizobium*, *Neorhizobium*, *Pararhizobium*, *Rhizobium*, *Pantoea*, *Candidatus* Phytoplasma, *Cladosporium*, *Fusarium*, and *Exophiala* were the major genera that contributed to differentiate the rhizosphere and endosphere communities.

### 3.3 Analysis of plant compartment/niche and climate indicator species

Indicator species analysis identified the bacterial and fungal taxa that significantly benchmark each plant compartment/niche and/or climate. We detected 35 bacterial indicator OTUs (Table 2) belonging to 13 families and 37 fungal OTUs (Table 3) belonging to 19 families. Only six fungal OTUs were significantly associated (*p* < 0.05) with future climate conditions and belonged to Plectosphaerellaceae, Stachybotryaceae, Helotiales, and Hypocreales, which colonized the root and leaf/stem endospheres.

**TABLE 2:** Indicator species analysis for bacterial operational taxonomic units (OTUs) across all samples and in the leaf/stem and root endospheres. No indicator OTUs were detected in the flower endosphere

Community	Treatment	Indicator OTU	Component A	Component B	Indicator value	*p*‐value	Indicator species	Family
All samples	Leaf/stem	OTU091	0.9622	1	0.981	0.001	*Rathayibacter_tritici*	Microbacteriaceae
		OTU061	0.9435	1	0.971	0.001	*Rhodococcus cercidiphylli*	Nocardiaceae
		OTU118	0.9016	1	0.95	0.001	*Burkholderiaceae*	Burkholderiaceae
		OTU119	0.8926	1	0.945	0.001	*Curtobacterium flaccumfaciens*	Microbacteriaceae
		OTU087	0.9801	0.9	0.939	0.001	*Sphingobium*	Sphingomonadaceae
		OTU070	0.8485	1	0.921	0.001	*Sphingomonas*	Sphingomonadaceae
		OTU068	0.8465	1	0.92	0.001	*Pseudomonas*	Pseudomonadaceae
		OTU156	0.8376	1	0.915	0.001	*Devosia*	Devosiaceae
		OTU044	0.8235	1	0.907	0.001	*Brevundimonas intermedia*	Caulobacteraceae
		OTU139	0.7944	1	0.891	0.002	*Actinomycetospora*	Pseudonocardiaceae
		OTU047	0.7792	0.9	0.837	0.016	*Nocardioides*	Nocardioidaceae
		OTU120	0.8713	0.8	0.835	0.018	*Pseudomonas*	Pseudomonadaceae
All samples	Root	OTU079	1	1	1	0.001	*Allorhizobium‐Neorhizobium‐Pararhizobium‐Rhizobium*	Rhizobiaceae
		OTU145	1	1	1	0.001	*Allorhizobium‐Neorhizobium‐Pararhizobium‐Rhizobium*	Rhizobiaceae
		OTU064	0.9978	1	0.999	0.001	*Rhizobium leguminosarum*	Rhizobiaceae
		OTU003	0.9953	1	0.998	0.001	*Rhizobium leguminosarum*	Rhizobiaceae
		OTU073	0.975	1	0.987	0.001	*Alphaproteobacteria*	
		OTU051	0.9545	1	0.977	0.001	*Kribbella*	Nocardioidaceae
		OTU031	0.9533	1	0.976	0.001	*Bradyrhizobium*	Xanthobacteraceae
		OTU084	0.9328	1	0.966	0.001	*Gammaproteobacteria*	
		OTU077	0.9296	1	0.964	0.001	*Mesorhizobium*	Rhizobiaceae
		OTU107	0.9086	1	0.953	0.001	*Pseudonocardia*	Pseudonocardiaceae
		OTU034	0.9077	1	0.953	0.003	*Ensife meliloti*	Rhizobiaceae
		OTU085	0.9036	1	0.951	0.001	*Phyllobacterium*	Rhizobiaceae
		OTU036	0.8996	1	0.948	0.001	*Rhizobacter*	Burkholderiaceae
		OTU098	0.9965	0.9	0.947	0.001	*Allorhizobium‐Neorhizobium‐Pararhizobium‐Rhizobium*	Rhizobiaceae
		OTU045	0.8519	1	0.923	0.001	*Aeromicrobium*	Nocardioidaceae
		OTU112	0.9231	0.9	0.911	0.001	*Caulobacteraceae*	Caulobacteraceae
		OTU060	0.8636	0.9	0.882	0.001	*Arenimonas*	Xanthomonadaceae
		OTU076	0.8421	0.9	0.871	0.001	*Sphingomonas*	Sphingomonadaceae
		OTU063	0.7222	1	0.85	0.001	*Lapillicoccus*	Intrasporangiaceae
		OTU052	0.9	0.8	0.849	0.002	*Nitrosomonadaceae*	Nitrosomonadaceae
		OTU022	0.8875	0.8	0.843	0.015	*Solirubrobacterales*	
		OTU055	0.7368	0.7	0.718	0.018	*Gaiella*	Gaiellaceae
Leaf/stem	Future	OTU087	0.9328	1	0.966	0.003	*Sphingobium*	Sphingomonadaceae

**TABLE 3:** Indicator species analysis for fungal operational taxonomic units (OTUs) across all samples and each compartment

Community	Treatment	Indicator OTU	Component A	Component B	Indicator value	*p*‐value	Indicator species	Family
All samples	Future	OTU22	0.9017	0.6667	0.775	0.043	*Cadophora luteo‐olivacea*	Helotiales_fam_Incertae_sedis
		OTU72	0.9776	0.4667	0.675	0.046	*Gibellulopsis chrysanthemi*	Plectosphaerellaceae
		OTU46	0.9391	0.4667	0.662	0.037	*Myrothecium*	Stachybotryaceae
All samples	flower	OTU51	1	1	1	0.001	*Sclerotiniaceae*	Sclerotiniaceae
All samples	Leaf/stem	OTU12	0.9764	1	0.988	0.001	*Colletotrichum*	Glomerellaceae
		OTU16	0.9638	1	0.982	0.001	*Chaetosphaeronema*	Phaeosphaeriaceae
		OTU15	0.9002	1	0.949	0.004	*Alternaria alternata*	Pleosporaceae
		OTU53	0.9693	0.9	0.934	0.001	*Acremonium polychromum*	Hypocreales_fam_Incertae_sedis
		OTU43	0.8526	1	0.923	0.001	*Chaetosphaeronema*	Phaeosphaeriaceae
		OTU44	0.8464	1	0.92	0.006	*Vishniacozyma victoriae*	Bulleribasidiaceae
		OTU79	0.7753	1	0.881	0.002	*Vishniacozyma*	Bulleribasidiaceae
		OTU67	0.8274	0.9	0.863	0.004	*Articulospora*	Helotiaceae
		OTU42	0.9107	0.8	0.854	0.003	*Acremonium fusidioides*	Hypocreales_fam_Incertae_sedis
		OTU72	0.98	0.6	0.767	0.009	*G. chrysanthemi*	Plectosphaerellaceae
All samples	Root	OTU3	0.999	1	0.999	0.001	*Exophiala*	Herpotrichiellaceae
		OTU23	0.9974	1	0.999	0.001	*Ilyonectria macrodidyma*	Nectriaceae
		OTU33	0.9956	1	0.998	0.001	*Periconia*	Periconiaceae
		OTU5	0.9949	1	0.997	0.001	*Fusarium solani*	Nectriaceae
		OTU39	0.9862	1	0.993	0.001	*Exophiala*	Herpotrichiellaceae
		OTU24	0.9231	1	0.961	0.001	*Clonostachys rosea*	Bionectriaceae
		OTU11	0.9084	1	0.953	0.001	*Fusarium proliferatum*	Nectriaceae
		OTU50	0.9982	0.9	0.948	0.001	*Pleosporales*	unclassified Pleosporales
		OTU49	1	0.8	0.894	0.001	*Cistella albidolutea*	Hyaloscyphaceae
		OTU26	1	0.8	0.894	0.001	*Darksidea*	Lentitheciaceae
		OTU14	0.8812	0.9	0.891	0.001	*Fusarium*	Nectriaceae
		OTU28	0.9905	0.8	0.89	0.001	*Glarea*	Helotiaceae
		OTU25	0.8235	0.9	0.861	0.002	*Plenodomus biglobosus*	Leptosphaeriaceae
		OTU57	0.9034	0.8	0.85	0.002	*Tetracladium marchalianum*	Helotiaceae
		OTU63	0.7093	1	0.842	0.003	*Tetracladium*	Helotiaceae
		OTU69	1	0.7	0.837	0.001	*Helotiales*	
		OTU55	0.95	0.7	0.815	0.004	*Roussoella solani*	Thyridariaceae
		OTU47	0.9333	0.5	0.683	0.017	*Chaetomium angustispirale*	Chaetomiaceae
		OTU64	1	0.4	0.632	0.028	*Pleosporales*	
Leaf/stem	Future	OTU42	0.8214	1	0.906	0.001	*Acremonium fusidioides*	Hypocreales_fam_Incertae_sedis
		OTU60	0.9908	0.8	0.89	0.017	*Stachybotrys*	Stachybotryaceae
		OTU72	0.9576	0.8	0.875	0.010	*G. chrysanthemi*	Plectosphaerellaceae
Root	Future	OTU69	0.9461	0.8	0.87	0.01	*Helotiales*	

### 3.4 Potential function of *T*. *pratense* microbiome across different plant niches and climate conditions

**FIGURE 4:**
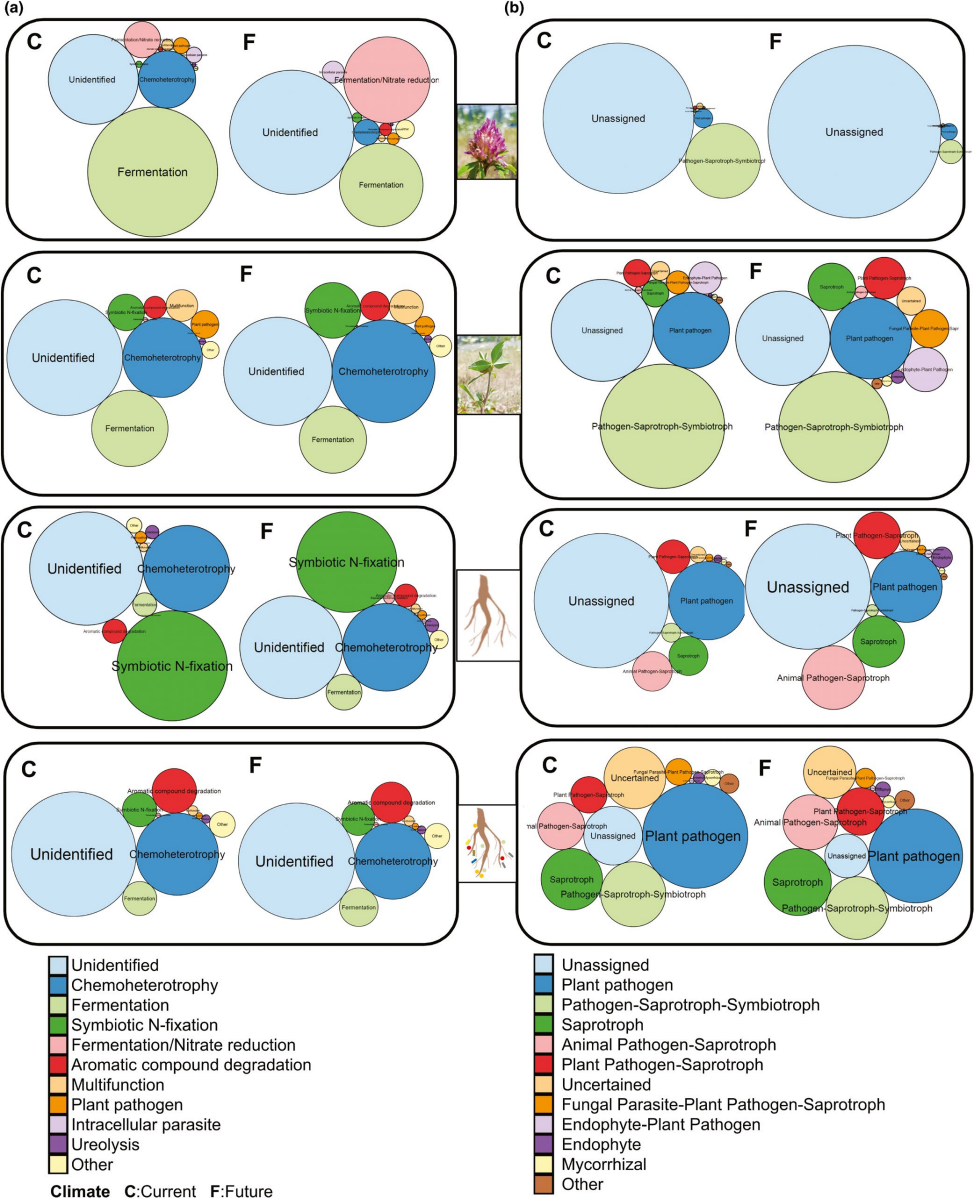
Functional characteristics of *Trifolium pratense* microbiome. Circle packing visualization of predicted trophic modes and functions of (a) bacterial and (b) fungal communities using FAPROTAX and FUNGuild databases for bacteria and fungi, respectively. The size of each circle represents the relative abundance of each function detected in each *T*. *pratense* compartment and climate condition. Climate: A = Current, F = Future

**FIGURE A5:**
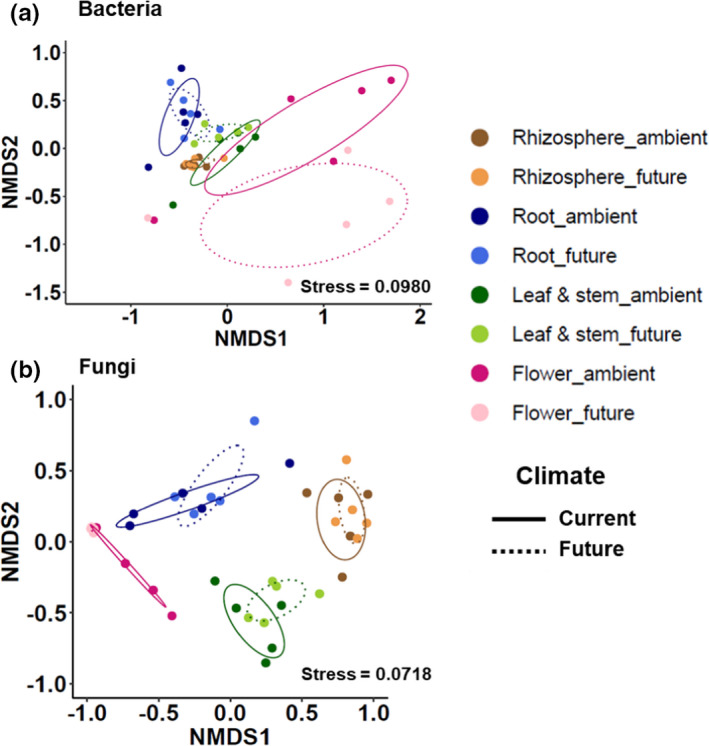
Nonmetric multidimensional scaling (NMDS) ordination of variation in bacterial and fungal trophic modes and specific functions of *Trifolium pratense* in each plant compartment under current and future climate conditions. *T*. *pratense* was grown in the grassland ecosystem. The plot is based on Bray–Curtis dissimilarities between microbial communities at the operation taxonomic unit level across 40 samples (permutations = 999). Samples (points) are shaded according to the plant compartment and climate. Ellipses indicate a 95% confidence interval surrounding each group

**FIGURE A6:**
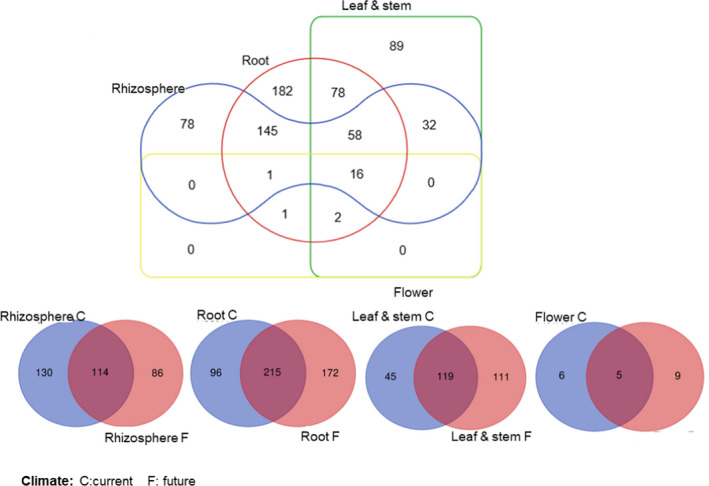
Venn diagrams showing the distribution of OTUs assigned as potential symbiotic N‐fixing bacteria in each plant compartment under current and future climate conditions

**FIGURE A7:**
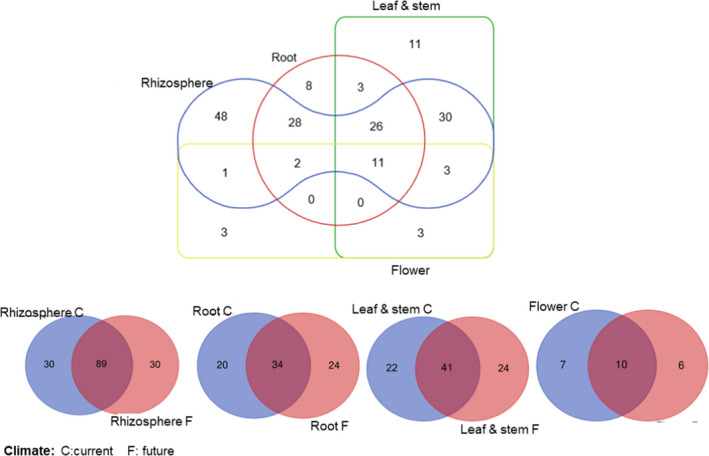
Venn diagrams showing the distribution of OTUs assigned as potential plant pathogenic fungi in each plant compartment under current and future climate conditions

FAPROTAX and FUNGuild were used to classify the bacterial and fungal OTU based on ecological functions to determine the microbial function distribution among the compartments of *T*. *pratense* and the climate conditions (Figure 4). NMDS analysis clustered the potential functional groups according to the plant compartment (Figure A5) for both bacteria (Bray–Curtis distances, *F* = 10.15 and *p* = 0.0001; Jaccard distance, *F* = 13.89 and *p* = 0.001) and fungi (Bray–Curtis distances, *F* = 45.00 and *p* = 0.001; Jaccard distance, *F* = 20.91 and *p* = 0.001) (Table A6). Climate conditions did not contribute to shaping the overall functional compositions. However, climate conditions affected the functions of the mycobiome of the leaf/stem endosphere. The relative abundances of saprotrophs (Mann–Whitney: *p* = 0.007), plant‐pathogen/saprotrophs (Mann–Whitney *U*; *p* = 0.031), and animal pathogen/saprotroph (Mann–Whitney *U*; *p* = 0.007) under future climate conditions were higher than that under current climate conditions. Additionally, we focused on the following two most important microbial functions: symbiotic N‐fixing bacteria and plant pathogenic fungi. In this study, 14 bacterial genera represented by 682 OTUs were assigned as symbiotic N‐fixing bacteria (Figure A6; Table A7), while 47 fungal genera represented by 177 OTUs were assigned as plant pathogens (Figure A7; Table A8). Interestingly, climate conditions did not affect these microbial functions (Table A9).

**FIGURE 5:**
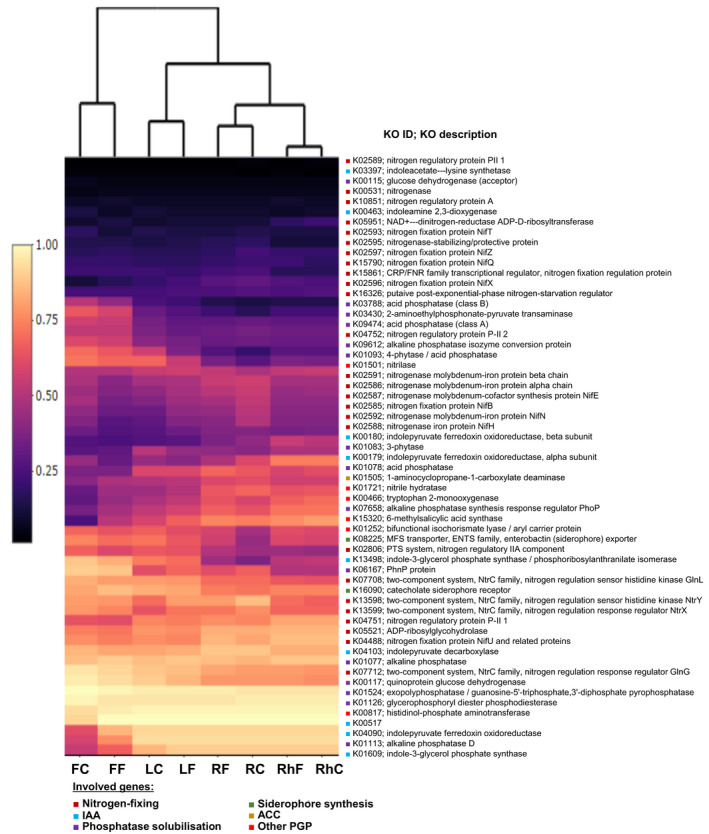
The heat map of normalized relative abundance of metabolic functional profiles of Kyoto Encyclopedia of Genes and Genomes (KEGG) orthologs (KOs) assigned to KEGG pathways involved in plant growth‐promoting (PGP) functions within *Trifolium pratense* bacterial microbiome. RhC, rhizosphere/current; RhF, rhizosphere/future; RC, root/current; RF, root/future; LC, leaf/stem/current; LF, leaf/stem/future; FC, flower/current; FF, flower/future; IAA, indole acetic acid; ACC, 1‐aminocyclopropane‐1‐carboxylate deaminase

### 3.5 Prediction of the metabolic functions of the bacterial community using Tax4Fun

**FIGURE A8:**
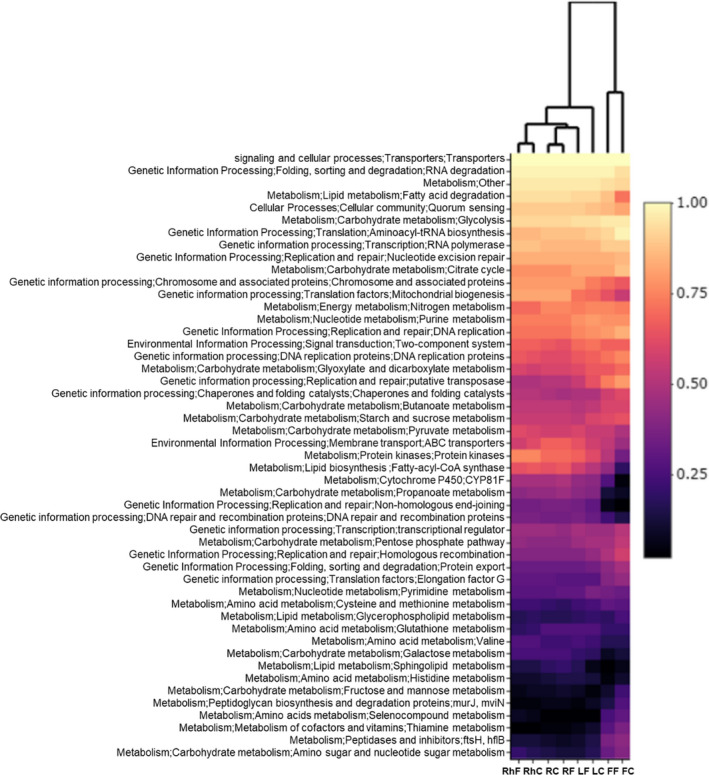
The heat map of normalized relative abundance of metabolic functional profiles of KOs assigned to KEGG pathways within in *T*. *pratense* bacterial microbiome using grouped into level‐3 functional categories. All of the functions of genes with an abundance >0.001%. RhC = rhizosphere/current, RhF = rhizosphere/future, RC = root/current, RF = root/future, LC = leaf & stem/current, LF = leaf & stem/future, FC = flower/current, FF = flower/future

The potential metabolic functional profiles of bacterial microbiomes were predicted based on the 16S rRNA genes of retrieved bacterial taxa using Tax4Fun according to the KEGG Ortholog groups (KOs) . The highly abundant metabolic genes (>0.001% sequence relative abundance) belonged to the following four categories: metabolism, genetic information processing, environmental information processing, and signaling, and cellular processes (Figure A8). Climate conditions did not affect the overall predicted metabolism of the bacterial communities (*F* = 0.73, *p* = 0.512). In contrast, the metabolic functions of bacteria in each compartment significantly varied (*F* = 13.01, *p* = 0.001) (Table A10). Additionally, the genes encoding plant growth‐promoting enzymes involved in biofertilization (N‐fixing: 28 genes, phosphate solubilization: 15 genes and siderophore synthesis: 2 genes) and biostimulation (indole acetic acid (IAA) production: 9 genes, 1‐aminocyclopropane‐1‐carboxylate (ACC) deaminase activity: 1 gene and general plant growth‐promoting traits: 6 genes) were predicted (Liang et al., 2020; Marasco et al., 2018) (Figure 5; Table A11). The climate conditions did not affect the composition of predicted functional genes involved in plant growth‐promoting traits (*F* = 0.97, *p* = 0.374; Table A12).

## 4 DISCUSSION

### 4.1 Red clover compartments/niches exhibit distinct microbial composition

In this study, the characterization of the red clover microbiome at the OTU and genus levels revealed that the individual plant compartments exhibited a unique microbial composition. This is consistent with the results of a recent study that reported a distinct microbial community in the flower and leaf epiphytes of *T*. *pratense* (Gaube et al., 2020). The bacterial composition is also reported to vary in different compartments of the model plants *Arabidopsis thaliana* (Bulgarelli et al., 2012), *Populus* spp. (Cregger et al., 2018), and *Medicago truncatula* (Brown et al., 2020), as well as those of the non‐model plants, such as *Myrtillocactus geometrizans* (Fonseca‐Garcia et al., 2016), *Opuntia robusta* (Fonseca‐Garcia et al., 2016), *Cycas panzhihuaensis* (Zheng & Gong, 2019), *Agave* spp (Coleman‐Derr et al., 2016), *Boechera stricta* (Wagner et al., 2016), and *Opuntia ficus*‐*indica* (Karray et al., 2020). The limited studies on the fungal phytobiomes have yielded similar results as the fungal composition was reported to be differentiated depending on plant compartments (Coleman‐Derr et al., 2016; Cregger et al., 2018; Fonseca‐Garcia et al., 2016; Gargouri et al., 2021; Zheng & Gong, 2019). The niche‐related differences in the microbiome composition can be attributed to variations in the microbial pools that invade different plant tissues through vertical transmission from seeds or horizontal transmission from soil and atmosphere (Cregger et al., 2018). The variations in the density of invading microbes and the unequal distributions of nutrients and oxygen among different plant tissues can also be a reason for microbial variations among different compartments (Vandenkoornhuyse et al., 2015).

Additionally, consistent with the results of other studies, the microbial diversity and richness varied between the plant compartments in this study. The analysis revealed that the microbial richness decreased from the rhizosphere to the endosphere tissues. This is due to the secretion of root exudates containing organic and amino acids, sugars, vitamins, hormones, and growth regulating substances in the rhizosphere, which promote microbial growth and colonization (Berg et al., 2016; Turner et al., 2013). In contrast, limited nutrients and available intercellular space in the plant endosphere limit microbial growth and colonization. The horizontal transfer of fungal communities from the rhizosphere to the endosphere was higher than that of bacterial communities. Among the rhizosphere fungal OTUs, 39% were transmitted to the root endosphere, 35% were shared with root and leaf/stem endospheres, and 6% were shared with all compartments. Similarly, among the rhizosphere bacterial OTUs, only 29% were transmitted to the root endosphere, 18% were shared with the root and leaf/stem endosphere, and only 4% were shared with all compartments. This suggested that the host genetic regulation of the bacterial composition is higher than that of the fungal composition and that the levels of host‐specific selection factors are high in the aboveground compartments. In contrast, the high level of specificity in the flower endosphere may indicate specific microbiome recruitment through air or pollinators (Vannette, 2020).

The analysis of the taxonomic composition of the clover microbiome in different compartments revealed that Actinobacteria and Sordariomycetes were the predominant microbes in the rhizosphere. In the root endosphere, Alphaproteobacteria (nitrogen‐fixing Rhizobia) was the predominant microbe, which was consistent with the results of a previous study on red clover (Hartman et al., 2017). Additionally, the root endosphere was less frequently colonized by other potential N‐fixing bacteria, such as *Bradyrhizobium*, *Devosia*, *Ensifer*, *Burkholderia*, *Mesorhizobium*, *Microvirga*, and *Phyllobacterium* (Table A7). Previous studies have reported that the roots of *Trifolium repens* and *Trifolium fragiferum* comprised *Rhizobium* as the predominant microbe with decreased abundance of rhizobia species, such as *Bradyrhizobium*, *Sinorhizobium*, and *Mesorhizobium* (Liu et al., 2007; Marilley & Aragno, 1999). The *T*. *pratense* root endosphere was enriched in OTUs of various genera, such as *Actinoplanes* and *Pseudomonas*. To the best of our knowledge, the microbial composition of the leaf/stem endosphere has not been previously investigated. In this study, the leaf/stem compartment predominantly comprised Gammaproteobacteria and Dothideomycetes. The species or strains of the most dominant bacterial and fungal genera in the root and leaf endosphere, such as *Actinoplanes* (Lazzarini et al., 2000), *Pseudonocardia* (Mangamuri et al., 2016), *Streptomyces* (Gouda et al., 2016), and *Cladosporium* (Gouda et al., 2016) are reported to synthesize medicinally important natural products. Flowers provide a unique habitat for microorganisms because of their ephemerality and anatomy, which form distinct micro‐niches (Aleklett et al., 2014). This study investigated the red clover inflorescence microhabitats (calyx, corolla, pistil, and stamen) as one unit. The microbiome of the flower predominantly comprised Gammaproteobacteria (*Pantoea*) and Mollicutes (*Candidatus* Phytoplasma), while the most prevalent fungal community members remained unidentified. A recent study on the seed‐borne endophytes of *T*. *pratense* revealed that the predominant bacterial taxa were Gammaproteobacteria (63% of relative sequences abundance, with a dominance of *Pantoea*) and unidentified fungi (70% of relative sequence abundance). This indicated that these taxa could be unique members of *T*. *pratense* flowers that are transmitted to the next generation via seeds. *Candidatus* Phytoplasma, which is the obligate bacterial pathogen of plant phloem, is transmitted through plant propagation materials and seeds, as well as by insect vectors (Kumari et al., 2019). In this study, microbial genera unique to the flower were detected, including the two insect symbionts, *Arsenophonus* and *Rickettsia*, which are transmitted by various arthropods (Caspi‐Fluger et al., 2012; Novakova et al., 2009). Thus, the plant served as a reservoir for the horizontal transmission of both bacterial genera.

### 4.2 Red clover harbors various beneficial microbes for plant growth and system sustainability

The analysis of the predicted bacterial functional genes showed various genes involved directly or indirectly in plant growth initiation and adaptation to climate changes. For example, this study predicted the presence of bacterial genes involved in siderophore synthesis that indirectly induce plant systemic resistance by enabling bacteria to compete with pathogens through the removal of iron from the environment (Bakker et al., 2007). Moreover, genes involved in the production of phytohormones, such as auxin, indole‐3‐acetic acid (IAA), and 1‐aminocyclopropane‐1‐carboxylic acid (ACC) that directly promote plant growth by enhancing cell division and differentiation or by lowering indigenous ethylene levels in the rhizosphere environment were predicted (Goren‐Saglam et al., 2020; Hayat et al., 2010; Van de Poel & Van Der Straeten, 2014). Additionally, IAA and ACC enable the host plants to adapt to abiotic environmental stress conditions (Ikram et al., 2018; Van de Poel & Van Der Straeten, 2014). In our study, *Pseudomonas*, *Streptomyces*, *and Pantoea* are three of the most abundantly detected genera in the rhizosphere and endosphere samples that are reported to promote plant growth and produce these bioactive compounds (Abbasi et al., 2019; Bakker et al., 2007; Jaemsaeng et al., 2018; Shariati et al., 2017). Furthermore, N‐fixing and phosphate solubilization genes, which are involved in enhancing plant growth and nutrient release to the soil and reduce the need for N and P fertilization (Hayat et al., 2010), were predicted. Therefore, *T*. *pratense* is considered one of the most important soil biofertilizer forage crops that contribute to system sustainability.

### 4.3 The impact of climate change on microbial community composition of *T*. *pratense*

**FIGURE A9:**
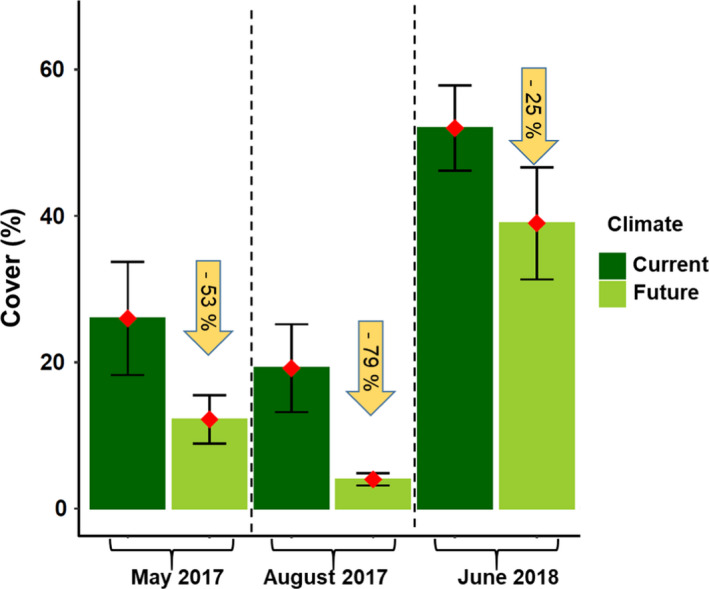
Percentage of *T*. *pratense* cover in the extensively managed meadow plots of the GCEF subjected to current and future climate scenarios. Error bars indicate the standard error; ♦ represent mean values. The sampling of rhizosphere and plant compartments for microbial analyses were performed in mid‐July 2018

Climate changes in terms of increasing temperature, summer drought, and altered precipitation patterns play a key role in shaping soil microbial communities (Mekala & Polepongu, 2019). However, few studies have investigated the effect of climatic conditions on plant‐associated microbiomes. Drought conditions obstruct root development leading to the limitation of water and nutrients uptakes by plants and the diminishment of plant biomass (Al‐Arjani et al., 2020; Hameed et al., 2014). In addition, severe drought may lead to over‐accumulation of reactive oxygen species that result in extensive plant cell damage and death (Cruz de Carvalho, 2008). Several plant‐associated microbes were found to contribute to drought stress tolerance in plants by carrying out various strategies. For instance, arbuscular mycorrhizal fungi‐plant associations lead to the induction of particular genes to elevated levels of expression such as P5CS involving in proline biosynthesis and genes coding for late embryogenesis abundant (LEA) proteins associated with ions and antioxidative stress system. Also, it regulates the abscisic acid (ABA) of plant content (Ahanger et al., 2014). Moreover, arbuscular mycorrhizal fungi could mitigate the negative effect of future climate conditions by altering the community composition and enhancing the richness of specific taxa (Wahdan et al., 2021). Recent studies (Gargouri et al., 2021; Karray et al., 2020) performed on the genus *Opuntia* revealed that bacterial and fungal plant microbiomes changed in the rhizosphere and root endosphere along a climatic aridity gradient. Moreover, they identified specific biomarker taxa for each bioclimatic zone. Additionally, increasing the aridity resulted in highly cohesive soil microbial‐root fungal networks. These microbial dynamics, biomarkers, and the highly correlative microbial networks could play a crucial role in the aridity stress and potentially promote the survival of *Opuntia*, one of the most xerophyte plants, across a wide range of arid zones (Gargouri et al., 2021). On the other hand, we have noticed that *T*. *pratense* resistance to cascading drought and rising soil temperature was limited. A marked reduction of *T*. *pratense* cover in the GCEF was detected after 4 years of growth under future climate conditions (unpublished results; Figure A9). In contrast to previous studies, our results revealed that *T*. *pratense* harbored a highly conserved microbiome that did not provide plasticity to the host to acquire desirable microbes or reconstruct the community structure as observed in the bacterial community. Fungal composition appeared to be more sensitive to environmental factors than bacterial composition, which was consistent with the results of previous studies (Coleman‐Derr et al., 2016; Cregger et al., 2018; Fonseca‐Garcia et al., 2016; Hacquard, 2016; Hamonts et al., 2018). We detected several dark septate endophytic fungal genera as indicators of future climate. Some of these indicators could be beneficial to plant growth and disease resistance, such as *Cadophora*, *Myrothecium*, and *Stachybotrys* (Banerjee et al., 2010; Busby et al., 2016; Yakti et al., 2019). However, these climate indicators are represented with few OTUs among the whole community.

## 5 CONCLUSIONS

To the best of our knowledge, this is the first study to examine the composition and functions of bacteria and fungi in the four compartments of the forage legume crop *T*. *pratense* under both current and future climate conditions. Although the *T*. *pratense* microbiomes did not differ at the community level, it is possible that the microbial communities changed at the genomic level that was not detected by our approach (16S and ITS sequencing data). Therefore, further studies on microbial functions involving the integration of the high‐resolution metagenome, metatranscriptome, and metaproteome approaches to unravel the entire gene expression and protein profiles of plant microbiota are required to provide more clear views of the microbial functions and their link to host performance. Moreover, a further controlled study is required to investigate the potential link between microbial composition and plant performance under future climate conditions.

## CONFLICT OF INTEREST

None declared.

## AUTHOR CONTRIBUTIONS

**Sara Fareed Mohamed Wahdan:** Data curation (lead); Formal analysis (lead); Investigation (lead); Methodology (lead); Software (equal); Visualization (lead); Writing‐original draft (lead). **Benjawan tanunchai:** Methodology (supporting); Writing‐review & editing (supporting). **Yuting Wu:** Methodology (equal); Writing‐review & editing (equal). **Chakriya Sansupa:** Methodology (supporting); Writing‐review & editing (supporting). **Martin Schädler:** Investigation (supporting); Project administration (lead); Writing‐review & editing (equal). **François Buscot:** Conceptualization (lead); Funding acquisition (lead); Project administration (lead); Resources (lead); Supervision (lead); Writing‐review & editing (lead). **Witoon Purahong:** Conceptualization (lead); Funding acquisition (lead); Investigation (supporting); Project administration (lead); Supervision (lead); Writing‐review & editing (lead). **Turki M. Dawoud:** Investigation (equal); Writing‐review & editing (supporting).

## ETHICS STATEMENT

None required.

## Data Availability

The bacterial 16S and fungal ITS2 raw read sequence datasets are available at the National Center for Biotechnology Information (NCBI) Sequence Read Archive (SRA) under BioProject accession number PRJNA680230: https://www.ncbi.nlm.nih.gov/bioproject/PRJNA680230

## APPENDIX 1

**TABLE A1:** Physicochemical properties of GCEF plots soil of grassland ecosystem under current and future climate conditions at the sampling time

Edaphic/climatic factor	Current climate	Future climate
pH	6.47 ± 0.18	6.53 ± 0.17
Organic matter (%)	5.18 ± 0.72	4.45 ± 0.41
P (ppm.)	129.86 ± 18.49	124.16 ± 14.33
CEC	8.64 ± 0.26	8.64 ± 0.3
K (m.e/100 g soil)	1.12 ± 0.36	1.19 ± 0.21
Na (m.e/100 g soil)	0.43 ± 0.25	0.54 ± 0.39
Ca (m.e/100 g soil)	21.08 ± 4.82	19.39 ± 3.83
Mg (m.e/100 g soil)	2.4 ± 0.03	2.55 ± 0.39
C/N	11.79 ± 2.25	10.25 ± 1.82
Precipitation (mm)	0.80 ± 0.23	0.82 ± 0.13
Soil temperature, mean value (°C)	22.34 ± 0.82	23.02 ± 0.37

Note

Values represent mean ± SD. The values did not differ significantly between ambient and future climate soils (*t*‐test, *p* > 0.05).

**TABLE A2:** Two‐way NPMANOVA and two‐way ANOSIM (Jaccard & Bray–Curtis dissimilarity matrix, permutations = 999) tested the influence of plant compartment, climate, and their interaction on *T*. *pratense* microbiome community composition based on OTU level

Source of variation/community	Jaccard distance				Bray–Curtis distances			
	Two‐way NPMANOVA		Two‐way ANOSIM		Two‐way NPMANOVA		Two‐way ANOSIM	
	Pseudo*F*	*p*	*R*	*p*	Pseudo*F*	*p*	*R*	*p*
Bacteria								
Plant compartment	8.684	**0.001**	0.765	**0.001**	10.386	**0.001**	0.656	**0.001**
Climate	0.991	0.382	0.005	0.471	1.386	0.201	−0.031	0.684
Plant compartment × climate	0.979	0.453	nd	nd	0.939	0.563	nd	nd
Fungi								
Plant compartment	7.123	**0.001**	0.941	**0.001**	24.912	**0.001**	0.792	**0.001**
Climate	1.583	*0.063*	0.206	**0.009**	1.366	0.244	0.074	0.104
Plant compartment × climate	1.072	0.307	nd	nd	1.116	0.335	nd	nd

Abbreviation: nd, not detected.

Significant values (*p* < 0.05) are indicated in bold, marginal significant (*p* < 0.1) values are indicated in italic.

**TABLE A3:** Pair‐wise post hoc test comparison using NPMANOVA on the Bray–Curtis similarity matrices in the total bacterial community to evaluate the ‘plant compartment’ effect

Compartment	F. Model	*R* ^2^	*p* adjusted sig.
Rh, R	10.437	0.367	0.001
Rh, L	10.802	0.375	0.001
Rh, F	8.538	0.321	0.001
R, L	8.154	0.311	0.001
R, F	7.937	0.306	0.001
L, F	4.452	0.198	0.001

Abbreviations: Rh, rhizosphere; R, root; L, leaf/stem; F, flower.

**TABLE A4:** Pair‐wise post hoc test comparison using NPMANOVA on the Bray–Curtis similarity matrices in the total fungal community to evaluate the ‘plant compartment’ effect

Compartment	F. Model	*R* ^2^	*p* adjusted sig.
F, L	45.209	0.715	0.001
F, Rh	48.685	0.730	0.001
F, R	10.259	0.363	0.001
L, Rh	20.290	0.529	0.001
L, R	19.500	0.520	0.001
Rh, R	16.556	0.479	0.001

Abbreviations: Rh, rhizosphere; R, root; L, leaf/stem; F, flower.

**TABLE A5:** Similarity percentages (SIMPER) analysis determines the genera contributions to the dissimilarity among compartments. In the upper part of the table, the compartment/niche pairwise comparison of average dissimilarity percentage has been reported. In the lower part, the overall top three genera contributing to the pairwise dissimilarity were listed

		Rhizosphere	Root	Leaf & stem	Flower
Bacterial community	Rhizosphere		68.32	69.13	91.48
	Root	*Allorhizobium*‐*Neorhizobium*‐*Pararhizobium*‐*Rhizobium* (28.29) *C*. *Phytoplasma* (6.63) *Actinoplanes* (6.43)		65.82	92.41
	Leaf & stem	*Pantoea* (14.23) *C*. *Phytoplasma* (13.54) *Pseudomonas* (5.63)	*Allorhizobium*‐*Neorhizobium*‐*Pararhizobium*‐*Rhizobium* (23.28) *Pantoea* (13.61) *C*.*_Phytoplasma* (12.48)		80.48
	Flower	*Pantoea* (23.44) *C*. *Phytoplasma* (8.12) *Serratia* (6.41)	*Allorhizobium*‐*Neorhizobium*‐*Pararhizobium*‐*Rhizobium* (22.91) *Pantoea* (20.43) *C*. *Phytoplasma* (15.31)	*Pantoea* (22.81) *C*. *Phytoplasma* (18.47) *Pseudomonas* (8.31)	
Fungal community	Rhizosphere		62.44	64.01	89.72
	Root	*Cladosporium* (15.42) *Fusarium* (10.29) *Gibellulopsis* (9.57)		83.9	96.03
	Leaf & stem	*Cladosporium* (25.12) *Fusarium* (11.70) *Exophiala* (8.00)	*Cladosporium* (39.21) *Exophiala* (9.04) *Fusarium* (8.70)		79.37
	Flower	*Cladosporium* (14.94) *Fusarium* (14.33) *Exophiala* (9.68)	*Exophiala* (18.50) *Fusarium* (17.95) *Cladosporium* (16.84)	*Cladosporium* (47.26) *Plectosphaerella* (9.98) *Colletotrichum* (6.02)	

**TABLE A6:** NPMANOVA and ANOSIM (Jaccard & Bray–Curtis dissimilarity matrix, permutations = 999) tested the influence of plant compartments/niches as well as climate change on the microbial functional composition of *T*. *pratense* based on OTU level in each plant compartment separately

Source of variation/community	Jaccard distance				Bray–Curtis distances			
	Two‐way NPMANOVA		Two‐way ANOSIM		Two‐way NPMANOVA		Two‐way ANOSIM	
	Pseudo*F*	*p*	*R*	*p*	Pseudo*F*	*p*	*R*	*p*
Bacteria								
Plant compartment	13.897	**0.001**	0.544	**0.001**	10.153	**0.0001**	0.472	**0.0001**
Climate	1.519	0.163	0.093	*0.053*	1.102	0.332	−0.0345	0.681
Plant compartment × climate	1.582	*0.095*	nd	nd	1.190	0.282	nd	nd
Fungi								
Plant compartment	20.912	**0.001**	0.625	**0.001**	45.007	**0.001**	0.864	**0.001**
Climate	1.357	0.231	0.026	0.308	2.388	*0.093*	0.138	**0.021**
Plant compartment × climate	0.770	0.641	nd	nd	1.332	0.255	nd	nd

Abbreviation: nd, not detected.

Significant values (*p* < 0.05) are indicated in bold, marginal significant (*p* < 0.1) values are indicated in italic.

**TABLE A7:** OTUs number of each potential symbiotic N‐fixing bacteria detected in each *T*. *pratense* compartment under current and future climate conditions

Genera	Current climate				Future climate			
	Rhizosphere	Root	Leaf/stem	Flower	Rhizosphere	Root	Leaf/stem	Flower
*Allorhizobium‐Neorhizobium‐Pararhizobium‐Rhizobium*	110	207	84	3	71	222	100	4
*Aminobacter*	0	1	0	0	0	1	1	0
*Bradyrhizobium*	40	11	3	1	39	37	6	1
*Burkholderia‐Caballeronia‐Paraburkholderia*	6	4	0	0	13	10	0	0
*Devosia*	18	27	23	2	14	32	45	1
*Ensifer*	3	3	1	0	2	7	2	1
*Mesorhizobium*	10	8	4	2	6	14	8	0
*Methylobacterium*	5	5	16	1	5	10	21	3
*Microvirga*	30	6	3	1	27	10	8	1
*Ochrobactrum*	0	1	1	0	0	2	0	0
*Phyllobacterium*	6	4	3	0	8	7	5	0
*Rhizobium*	15	33	23	1	13	31	32	2
*Rhodopseudomonas*	0	1	1	0	1	2	1	1
*Shinella*	1	0	2	0	1	2	1	0

**TABLE A8:** OTUs number of each potential plant pathogenic fungi detected in each *T*. *pratense* compartment under current and future climate conditions

Genera	Current climate				Future climate			
	Rhizosphere	Root	Leaf/stem	Flower	Rhizosphere	Root	Leaf/stem	Flower
*Stemphylium*	2	0	3	0	0	0	2	0
*Alternaria*	5	2	7	2	6	3	9	2
*Boeremia*	2	1	3	1	2	1	5	0
*Fusarium*	23	16	9	3	20	18	6	4
*Ilyonectria*	6	4	0	1	3	1	0	0
*Nectria*	1	1	0	0	1	0	0	0
*Neoascochyta*	1	0	0	0	0	0	1	1
*Periconia*	1	1	1	0	1	0	1	0
*Acremonium*	1	0	1	0	1	1	1	0
*Ascochyta*	2	0	2	0	2	0	1	0
*Bipolaris*	0	0	0	0	1	0	1	0
*Botrytis*	1	0	0	0	1	1	0	1
*Cercospora*	1	1	1	1	1	1	1	1
*Clonostachys*	8	4	2	1	7	3	1	0
*Colletotrichum*	0	0	1	0	2	0	0	0
*Curvularia*	0	0	0	0	1	0	0	0
*Cylindrocarpon*	1	0	0	0	1	0	0	0
*Dendryphion*	1	1	0	0	1	1	1	0
*Devriesia*	0	0	0	0	1	1	0	0
*Didymella*	1	3	1	1	2	2	2	1
*Edenia*	2	2	1	0	2	1	0	0
*Eocronartium*	4	0	0	0	4	0	0	0
*Erysiphe*	1	0	1	1	1	0	1	1
*Erythricium*	1	0	0	0	1	1	1	0
*Gaeumannomyces*	0	0	0	0	1	0	0	0
*Gibberella*	11	5	9	0	11	3	5	0
*Gibellulopsis*	14	2	3	0	12	2	2	1
*Golovinomyces*	0	0	0	1	0	0	0	1
*Itersonilia*	1	1	1	0	1	0	0	0
*Laetisaria*	0	0	0	0	1	0	0	0
*Lectera*	1	1	1	0	1	0	1	0
*Leptosphaeria*	1	1	0	0	1	1	0	0
*Magnaporthiopsis*	1	0	0	0	1	0	0	0
*Monosporascus*	0	1	0	0	2	1	0	0
*Mycoleptodiscus*	0	0	0	0	0	1	0	0
*Mycosphaerella*	0	0	0	0	1	0	0	0
*Oculimacula*	0	0	0	0	0	0	1	0
*Plectosphaerella*	15	4	11	0	16	9	15	0
*Podosphaera*	0	0	0	1	0	0	0	1
*Powellomyces*	2	0	0	0	1	0	0	0
*Pyrenophora*	0	0	1	1	0	0	0	1
*Ramularia*	1	0	0	0	1	0	1	0
*Septoria*	1	0	1	0	1	0	1	0
*Slopeiomyces*	3	2	1	0	1	2	0	0
*Stagonosporopsis*	0	0	0	0	1	1	2	0
*Thanatephorus*	2	1	0	0	2	2	2	0
*Volutella*	1	0	0	0	0	0	0	0
Ustilaginaceae unclassified	0	0	2	2	2	1	1	0
Sclerotiniaceae unclassified	0	0	0	1	0	0	0	1

**TABLE A9:** NPMANOVA and ANOSIM (Jaccard dissimilarity matrix, permutations = 999) tested the influence of plant compartments/niches as well as climate change on the microbial functional composition of potential N‐fixing bacteria and pathogenic fungi

Source of variation/community	Jaccard distance			
	Two‐way NPMANOVA		Two‐way ANOSIM	
	Pseudo*F*	*p*	*R*	*p*
Potential N‐fixing bacteria				
Plant compartment	3.95	**0.001**	0.60	**0.0001**
Climate	0.96	0.50	0.025	0.320
Plant compartment × climate	1.00	0.43	nd	nd
Potential plant pathogenic fungi				
Plant compartment	8.69	**0.001**	0.903	**0.001**
Climate	1.51	0.101	0.019	0.378
Plant compartment × climate	1.01	0.397	nd	nd

Abbreviation: nd, not detected.

Significant values (*p* < 0.05) are indicated in bold.

**TABLE A10:** NPMANOVA and ANOSIM (Bray–Curtis dissimilarity matrix, permutations = 999) tested the influence of plant compartments/niches, as well as climate change on bacterial community, predicted metabolic functional attributes using Tax4Fun

Source of variation/community	Bray–Curtis distances			
	Two‐way NPMANOVA		Two‐way ANOSIM	
	Pseudo*F*	*p*	*R*	*p*
Bacteria				
Plant compartment	**13.01**	**0.001**	0.47	**0.001**
Climate	0.73	0.512	−0.09	0.961
Plant compartment × climate	0.4524	0.866	nd	nd

Abbreviation: nd, not detected.

Significant values (*p* < 0.05) are indicated in bold.

**TABLE A11:** List of the selected KEGG enzyme‐encoding gene for plant growth‐promoting traits involved in biofertilization (nitrogen fixation, phosphate solubilization, and siderophore synthesis) and biostimulation (indole acetic acid (IAA) production, 1‐aminocyclopropane‐1‐carboxylate (ACC) deaminase activity, and general plant growth‐promoting traits). All data were extracted from the Kyoto Encyclopaedia for Genes and Genomes (KEGG) database www.genome.jp/kegg/

Function	gene
N‐fixation	K00531; nitrogenase [EC:1.18.6.1]
	K02585; nitrogen fixation protein NifB
	K02586; nitrogenase molybdenum‐iron protein alpha chain [EC:1.18.6.1]
	K02587; nitrogenase molybdenum‐cofactor synthesis protein NifE
	K02588; nitrogenase iron protein NifH [EC:1.18.6.1]
	K02589; nitrogen regulatory protein P‐II 1
	K02590; nitrogen regulatory protein P‐II 2
	K02591; nitrogenase molybdenum‐iron protein beta chain [EC:1.18.6.1]
	K02592; nitrogenase molybdenum‐iron protein NifN
	K02593; nitrogen fixation protein NifT
	K02595; nitrogenase‐stabilizing/protective protein
	K02596; nitrogen fixation protein NifX
	K02597; nitrogen fixation protein NifZ
	K02806; PTS system, nitrogen regulatory IIA component [EC:2.7.1.69]
	K04488; nitrogen fixation protein NifU and related proteins
	K04751; nitrogen regulatory protein P‐II 1
	K04752; nitrogen regulatory protein P‐II 2
	K05521; ADP‐ribosylglycohydrolase [EC:3.2.‐.‐]
	K05951; NAD+‐‐‐dinitrogen‐reductase ADP‐D‐ribosyltransferase [EC:2.4.2.37]
	K07708; two‐component system, NtrC family, nitrogen regulation sensor histidine kinase GlnL [EC:2.7.13.3]
	K07712; two‐component system, NtrC family, nitrogen regulation response regulator GlnG
	K10851; nitrogen regulatory protein A
	K13598; two‐component system, NtrC family, nitrogen regulation sensor histidine kinase NtrY [EC:2.7.13.3]
	K13599; two‐component system, NtrC family, nitrogen regulation response regulator NtrX
	K15790; nitrogen fixation protein NifQ
	K15861; CRP/FNR family transcriptional regulator, nitrogen fixation regulation protein
	K16326; CRP/FNR family transcriptional regulator, putative post‐exponential‐phase nitrogen‐starvation regulator
Siderophore synthesis	K08225; MFS transporter, ENTS family, enterobactin (siderophore) exporter
	K16090; catecholate siderophore receptor
Indole acetic acid (IAA) production	K01609; indole‐3‐glycerol phosphate synthase [EC:4.1.1.48]
	K00517; [EC:1.14.‐.‐]
	K03397; indoleacetate‐‐‐lysine synthetase [EC:6.3.2.20]
	K04103; indolepyruvate decarboxylase [EC:4.1.1.74]
	K04090; indolepyruvate ferredoxin oxidoreductase [EC:1.2.7.8]
	K13498; indole‐3‐glycerol phosphate synthase / phosphoribosylanthranilate isomerase [EC:4.1.1.48 5.3.1.24]
	K00179; indolepyruvate ferredoxin oxidoreductase, alpha subunit [EC:1.2.7.8]
	K00180; indolepyruvate ferredoxin oxidoreductase, beta subunit [EC:1.2.7.8]
	K00463; indoleamine 2,3‐dioxygenase [EC:1.13.11.52]
	K04103; indolepyruvate decarboxylase [EC:4.1.1.74]
1‐aminocyclopropane‐1‐carboxylate (ACC) activity	K01505; 1‐aminocyclopropane‐1‐carboxylate deaminase [EC:3.5.99.7]
General plant growth‐promoting traits	K15320; 6‐methylsalicylic acid synthase [EC:2.3.1.165]
	K01252; bifunctional isochorismate lyase / aryl carrier protein [EC:3.3.2.1]
	K01501; nitrilase [EC:3.5.5.1]
	K00466; tryptophan 2‐monooxygenase [EC:1.13.12.3]
	K01721; nitrile hydratase [EC:4.2.1.84]
	K00817; histidinol‐phosphate aminotransferase [EC:2.6.1.9]
Phosphate solubilization	K00117; quinoprotein glucose dehydrogenase [EC:1.1.5.2]
	K00115; glucose dehydrogenase (acceptor) [EC:1.1.99.10]
	K01083; 3‐phytase [EC:3.1.3.8]
	K01093; 4‐phytase / acid phosphatase [EC:3.1.3.26 3.1.3.2]
	K01078; acid phosphatase [EC:3.1.3.2]
	K01093; 4‐phytase / acid phosphatase [EC:3.1.3.26 3.1.3.2]
	K03788; acid phosphatase (class B) [EC:3.1.3.2]
	K09474; acid phosphatase (class A) [EC:3.1.3.2]
	K09612; alkaline phosphatase isozyme conversion protein [EC:3.4.11.‐]
	K01077; alkaline phosphatase [EC:3.1.3.1]
	K01113; alkaline phosphatase D [EC:3.1.3.1]
	K07658; two‐component system, OmpR family, alkaline phosphatase synthesis response regulator PhoP
	K06167; PhnP protein
	K01524; exopolyphosphatase / guanosine‐5'‐triphosphate,3'‐diphosphate pyrophosphatase [EC:3.6.1.11 3.6.1.40]
	K03430; 2‐aminoethylphosphonate‐pyruvate transaminase [EC:2.6.1.37]
	K01126; glycerophosphoryl diester phosphodiesterase [EC:3.1.4.46]

**TABLE A12:** NPMANOVA and ANOSIM (Bray–Curtis dissimilarity matrix, permutations = 999) tested the influence of plant compartments/niches, as well as climate change on bacterial community, predicted metabolic functional involved in plant growth‐promoting (PGP) traits using Tax4Fun

Source of variation/community	Bray–Curtis distances			
	Two‐way NPMANOVA		Two‐way ANOSIM	
	Pseudo*F*	*p*	*R*	*p*
Bacteria				
Plant compartment	**14.76**	**0.001**	0.45	**0.001**
Climate	0.97	0.374	−0.05	0.853
Plant compartment × climate	0.67	0.671	nd	nd

Abbreviation: nd, not detected.

Significant values (*p* < 0.05) are indicated in bold.
